# PIEZO1 is Required for Acute Myeloid Leukemia Progression and Leukemia Stem Cell Maintenance via HIF1A‐SLC7A11 Axis‐Mediated Ferroptosis Defense

**DOI:** 10.1002/advs.75648

**Published:** 2026-05-10

**Authors:** Tiantian Zhang, Ziyan Cao, Kexin Gao, Shuxin Yao, Yanbing Zheng, Manman Cui, Rong Yin, Guoqiang Han, Jin Hu, Chengyi Wang, Haojian Zhang

**Affiliations:** ^1^ State Key Laboratory of Oral & Maxillofacial Reconstruction and Regeneration Hubei Key Laboratory of Stomatology Key Laboratory of Oral Biomedicine Ministry of Education School & Hospital of Stomatology Wuhan University Wuhan China; ^2^ Frontier Science Center For Immunology and Metabolism Medical Research Institute Wuhan University Wuhan China; ^3^ Department of Hematology Zhongnan Hospital Wuhan University Wuhan China; ^4^ Department of Pediatrics Fujian Children's Hospital (Fujian Branch of Shanghai Children's Medical Center) College of Clinical Medicine For Obstetrics & Gynecology and Pediatrics Fujian Medical University Fuzhou China; ^5^ Taikang Center for Life and Medical Sciences Wuhan University Wuhan China

**Keywords:** acute myeloid leukemia, ferroptosis, leukemia stem cells, metabolism, PIEZO1

## Abstract

Acute myeloid leukemia (AML) is a highly aggressive hematologic malignancy that arises from leukemia stem cells (LSCs) transformed from normal hematopoietic stem progenitor cells (HSPCs). Compared to normal HSPCs, LSCs acquire various adaptive properties that enable their survival and regenerative capacity under environmental challenges. However, the key regulators underlying these adaptative features in AML LSCs remain largely unknown. Here, the mechanosensor PIEZO1 as a key factor in promoting acute myeloid leukemia progression and maintaining LSC stemness by HIF1A‐SLC7A11 axis‐mediated ferroptosis defense is uncovered. PIEZO1 deletion impairs cystine uptake and induces ROS and lipid peroxidation, causing ferroptosis of leukemia cells. Mechanistically, HIF1A mediates the function of PIEZO1 by transcriptionally regulating the expression of SLC7A11 and SLC3A2. Further, inhibition of PIEZO1 and SLC7A11 suppresses LSC function and contributes to AML treatment. In summary, this findings reveal that LSCs control cystine uptake through the PIEZO1‐HIF1A‐SLC7A11 axis to defend against ferroptosis, representing a unique vulnerability of LSCs.

## Graphical abstract

This study identifies the mechanosensor PIEZO1 as a key factor in promoting acute myeloid leukemia progression and uncovers a ferroptosis defense system mediated by PIEZO1‐HIF1A‐SLC7A11 axis that is essential for leukemia stem cell maintenance. This ferroptotic defense system may represent a unique vulnerability for leukemia stem cells, providing a therapeutic strategy in treating acute myeloid leukemia.

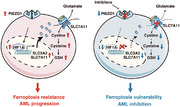

## 1 Introduction

Acute myeloid leukemia (AML) represents a highly aggressive and fatal hematologic malignancy featuring aberrant differentiation and abnormal expansion of immature myeloid precursors [1]. Although significant advances have been achieved in AML treatment in recent decades, the 5‐year survival rate of AML patients remains relatively low, at approximately 30%. Therefore, a more profound insight into AML pathogenesis and the identification of novel therapeutic targets are urgently needed. It is well established that most hematologic malignancies arise from the progressive buildup of genetic or epigenetic alterations within hematopoietic stem/progenitor cells (HSPCs) [2, 3]. These alterations drive the transformation of HSPCs into leukemia stem cells (LSCs), thereby catalyzing AML pathogenesis [4, 5]. Importantly, LSCs exhibit resistance to chemotherapy and are major contributors to disease relapse [3, 5, 6, 7]. Thus, investigating LSC vulnerability may provide potential therapeutic strategies for curing AML.

LSCs acquire unique adaptations essential for the cellular transition from normal HSCs to a malignant state [8, 9, 10]. Understanding these adaptations could offer new insights into the selective vulnerabilities of LSCs. Our previous studies have revealed significant alterations in RNA m^6^A methylome during leukemogenesis and linked chromatin state dynamics to the expression regulation of m^6^A modifiers [8, 9]. Additionally, while normal HSCs rely primarily on glycolysis, human LSCs depend on oxidative phosphorylation (OXPHOS) for survival [11, 12, 13]. This metabolic difference reflects mitochondrial adaptations that confer key properties to LSCs by dynamically reprogramming cellular bioenergetics and metabolism [10]. Metabolic reprogramming in LSCs also influences their preference for different nutrients and metabolites that fuel energy metabolism. For example, human AML LSCs exhibit altered uptake of amino acids, fatty acids, and lipids [13, 14]. A recent study showed that AML is frequently associated with decreased heme levels, and that inhibition of heme biosynthesis can trigger cuproptosis [15]. To date, targeting LSC metabolism remains a promising strategy for improving clinical outcomes for many AML patients [13]. For instance, inhibitors of metabolic enzymes IDH1/2, such as ivosidenib (IDH1) and enasidenib (IDH2), are approved therapies for relapsed or refractory AML patients harboring the respective mutations [16]. Therefore, leveraging the metabolic properties of LSCs may lead to more effective and promising therapeutic regimens, and a deeper understanding of LSC metabolism is crucial for improving outcomes in AML patients.

Stem cells sense and react to biochemical and physical signals to preserve tissue homeostasis and regeneration [17]. Similarly, both HSCs and LSCs inhabit the complex stromal niche of the bone marrow, which provides a variety of factors that tightly regulate stem cell fate. Studies have shown that the bone marrow niche is altered in AML to promote disease progression. Interestingly, a recent study uncovered that LSCs exhibit unique mechanical properties, being smaller and mechanically softer [18], suggesting a distinct mechanical adaptation in LSCs. Mechanosensor proteins PIEZO1 and PIEZO2 are activated in response to mechanical stimuli and are critically involved in mechanotransduction by regulating the interaction of the cells with the microenvironment and controlling cellular functions [17, 19]. However, the role of PIEZO mechanosensitive channels in LSCs is still unclear. Here, we demonstrate that PIEZO1 is essential for maintaining LSC function and AML progression through the PIEZO1‐HIF1A‐SLC7A11 axis‐mediated ferroptosis defense, thereby revealing a unique vulnerability of LSCs.

## 2 Results

### 2.1 PIEZO1 is Required for Human AML Cells Survival

**FIGURE 1:**
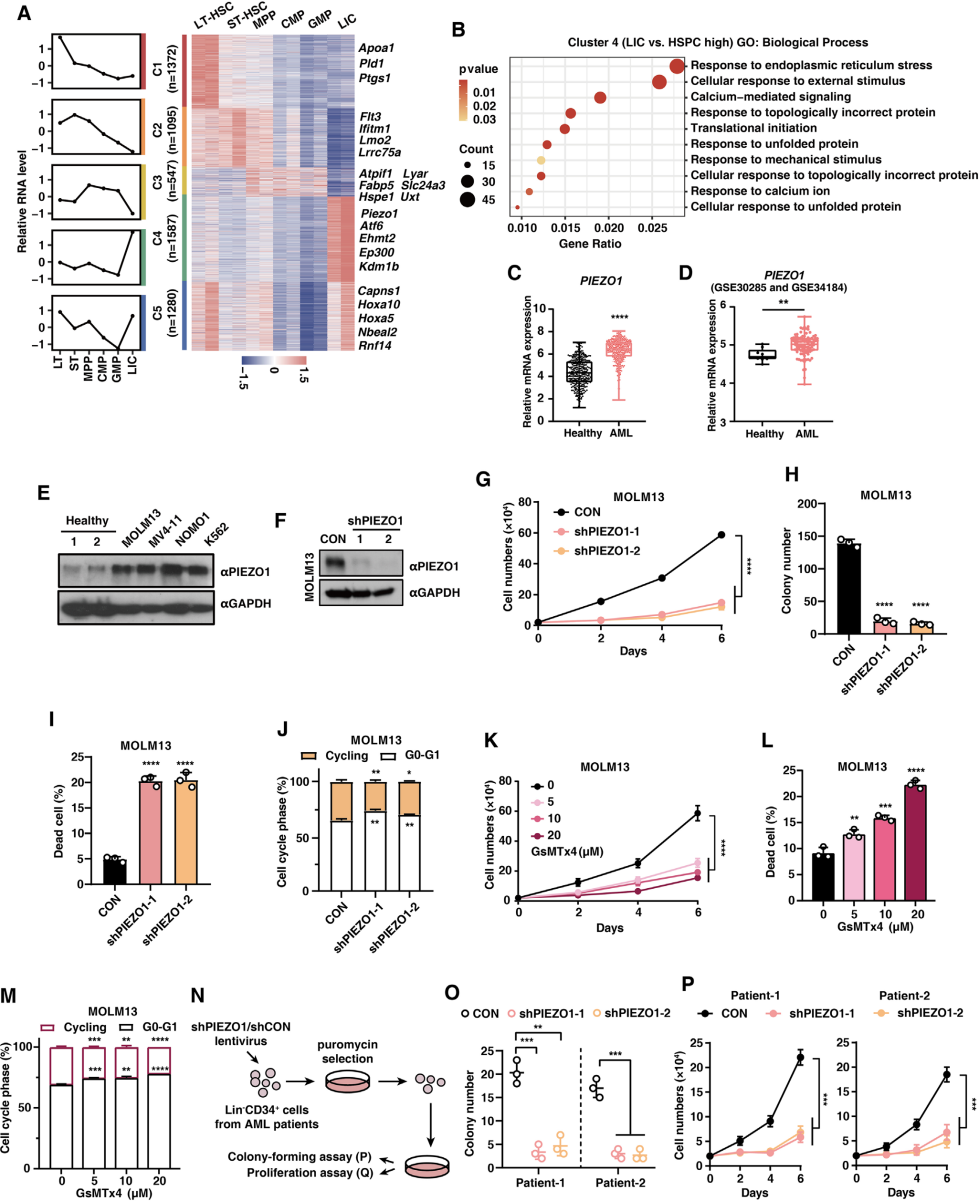
Elevated expression of PIEZO1 Is Required for Survival of Human Myeloid Leukemia Cells. (A) Heatmap showing the mRNA expression levels of 5881 differential gene expression in HSPCs and LICs (Right). The trend of mRNA dynamics for these 5 clusters (Left). LT‐HSC, long‐term hematopoietic stem cells; ST‐HSC, short‐term hematopoietic stem cell; MPP, multipotent progenitor; CMP, common myeloid progenitor; GMP, granulocyte‐macrophage progenitor; LIC, leukemia‐initiating cell. Data from GSE165863 and GSE210280. (B) GO enrichment analysis of genes in cluster 4. (C) Expression of PIEZO1 mRNA in AML patient samples, compared with healthy controls. Data combined from TCGA (*n* = 173), GTEx (*n* = 337), and TARGET (*n* = 196) database. Expression values are log2(FPKM+0.001). Box plots show median, quartiles, and whiskers. (D) Comparison of PIEZO1 expression in samples of AML patients (n = 97) and healthy donors (*n* = 9) (GSE30285 and GSE34184). (E) Western blot assay of PIEZO1 expression in different human myeloid leukemia cell lines and bulk BM mononuclear cells from healthy donors (*n* = 2). GAPDH served as the loading control. (F) Western blot assay showing knockdown efficiency of PIEZO1 in MOLM13 leukemia cells. GAPDH served as the loading control. (G–J) Growth curves (G), CFU assay (H), cell death analysis (I), cell cycle distribution (J) of MOLM13 leukemia cells after transduction with the indicated lentiviruses. *n* = 3 independent experiments, each performed in technical triplicates (G, I, J) or duplicates (H). For (G), two‐way ANOVA with Sidak's post‐hoc test. For (H–J), one‐way ANOVA with Tukey's post‐hoc test. (K–M) Growth curves (K), cell death analysis (L), cell cycle distribution (M) of MOLM13 leukemia cells treated with GsMTx4 in different doses (0, 5, 10, 20 µM). *n* = 3 independent experiments, each in triplicate. For (K), two‐way ANOVA with Dunnett's post‐hoc test. For (L,M), one‐way ANOVA with Dunnett's post‐hoc test. (N) Experimental scheme for (O,P). (O) CFU assay of AML patient‐derived Lin^−^CD34^+^ cells after transduction with the indicated lentiviruses. Lin, lineage. independent patient samples (each measured in duplicate). (P) Growth curves of AML patient‐derived Lin^−^CD34^+^ cells after transduction with the indicated lentiviruses. *n* = 2 independent patient samples (each measured in triplicate). Two‐way ANOVA with Sidak's post‐hoc test. *p < 0.05, **p < 0.01, ***p < 0.001 and ****p < 0.0001. Unless otherwise specified in individual panels, all data are presented as mean ± SD (error bars), and statistical significance was determined using two‐tailed unpaired Student's t‐test (paired where indicated). Results are representative of two (O,P) and three (G–M) independent experiments.

To identify the key factors that regulate LSC function, we interrogated our in‐house generated and publicly available datasets [9, 20], and compared the transcriptional profiles of leukemia‐initiating cells (LICs) with different HSPC populations isolated from MLL‐AF9 induced AML mice and healthy control mice, respectively (Figure 1A). LICs (YFP^+^Lin^−^Sca‐1^−^c‐Kit^+^ cells) are enriched with leukemia stem cells. We identified 5881 differentially expressed genes (DEGs) across these different cell populations, and K‐mean clustering revealed 5 major clusters according to their expression dynamics. Interestingly, DEGs in cluster 4 displayed relative higher expression levels specifically in LICs but not normal HSPCs. We thus focused on this cluster, and gene ontology (GO) analysis demonstrated that these DEGs in cluster 4 showed predominant enrichment in biological processes, including response to endoplasmic reticulum stress, translational initiation, and response to unfolded protein (Figure 1B), which is in line with our previous studies [8, 10]. Interestingly, PIEZO1 is among the LIC‐specific gene cluster (Figure 1A). Compared to healthy controls, a significant upregulation of *PIEZO1* mRNA was observed in individuals with AML (Figure 1C,D; Figure S1A–C). Further analyzing publicly available single‐cell RNA‐seq data [21], We found that primitive AML cells from patients (including HSC‐like, Prog‐like, and GMP‐like malignant cells) exhibited higher expression of PIEZO1 than their normal counterparts (HSCs, Progs, and GMPs) from healthy bone marrow (Figure S1D). Similarly, higher PIEZO1 protein expression was detected in leukemia cell lines (e.g., MOLM13, MV4‐11, NOMO1, and K562) (Figure 1E). Thus, these findings suggest that PIEZO1 may contribute to AML pathogenesis.

We subsequently investigated the functional role of PIEZO1 in human leukemia. Short hairpin RNA (shRNA) targeting PIEZO1 (shPIEZO1#1 and #2) were used to efficiently knock down PIEZO1 expression, as shown by the significant decrease of PIEZO1 at both protein and mRNA levels (Figure 1F; Figure S1E,F). As expected, PIEZO1 knockdown substantially inhibited cell proliferation and clonogenicity in human leukemia cells MOLM13 and MV4‐11 (Figure 1G,H; Figure S1G,H). Furthermore, PIEZO1 knockdown significantly induced cell death, and modestly reduced the proportion of cycling cells (Figure 1I,J; Figure S1I–L). Further, we pharmaceutically inhibited PIEZO1 activity with its inhibitor GsMTx4 and Dooku1. Consistently, both GsMTx4 and Dooku1 treatment clearly decreased the proliferation, induced cell death, and inhibited the cycling of human leukemia cells (Figure 1K–M; Figure S1M–O). In addition, we assessed the function of PIEZO1 in primary Lin^−^CD34^+^ cells derived from AML patient, which are often regarded as the LSC‐enriched population (Figure 1N). Compared with the control samples, PIEZO1 knockdown markedly diminished proliferation and the clonogenic potential of these primary leukemia cells (Figure 1O,P; Figure S1P). Collectively, these data suggest that PIEZO1 serves a crucial function in human AML.

### 2.2 PIEZO1 Is Required for AML Progression and LSC Stemness in Mice

We next examined whether PIEZO1 is necessary for AML progression in mice. Initially, Piezo1 was silenced in primary leukemia cells isolated from a murine MLL‐AF9 AML model (Figure S2A). Knockdown efficiency was verified by quantitative mRNA analysis (Figure S2B). Consistent with expectations, Piezo1 knockdown suppressed the expansion and clonogenic capacity of leukemia cells (Figure S2C–E). We also transplanted these Piezo1‐knockdown leukemia cells into sublethally irradiated recipient mice. Knockdown of Piezo1 delayed AML progression in vivo, as shown by a reduced proportion of YFP^+^ leukemia cells in the peripheral blood (PB) and extended survival of engrafted mice (Figure S2F,G).

Additionally, we established *Piezo1^fl/fl^* conditional knockout mice and bred them with the *Mx1‐Cre* line to obtain *Mx1‐cre; Piezo1^fl/fl^* mice (Figure S2H). Experimental mice carrying the *Mx1‐cre; Piezo1^fl/fl^* genotype and their *Piezo1^fl/fl^* littermate controls, all at 8–10 weeks of age, were intraperitoneally injected with poly(I:C) (pIpC) 3 times at two‑day intervals. Effective deletion of Piezo1 was confirmed at the 4‐week time point after the final pIpC treatment (Figure S2I). To simplify, pIpC‐treated *Mx1‐cre; Piezo1^fl/fl^* mice were hereafter termed *Piezo1^cKO^* mice, while pIpC‐treated *Piezo1^fl/fl^* mice were designated as wild‐type (WT) controls.

**FIGURE 2:**
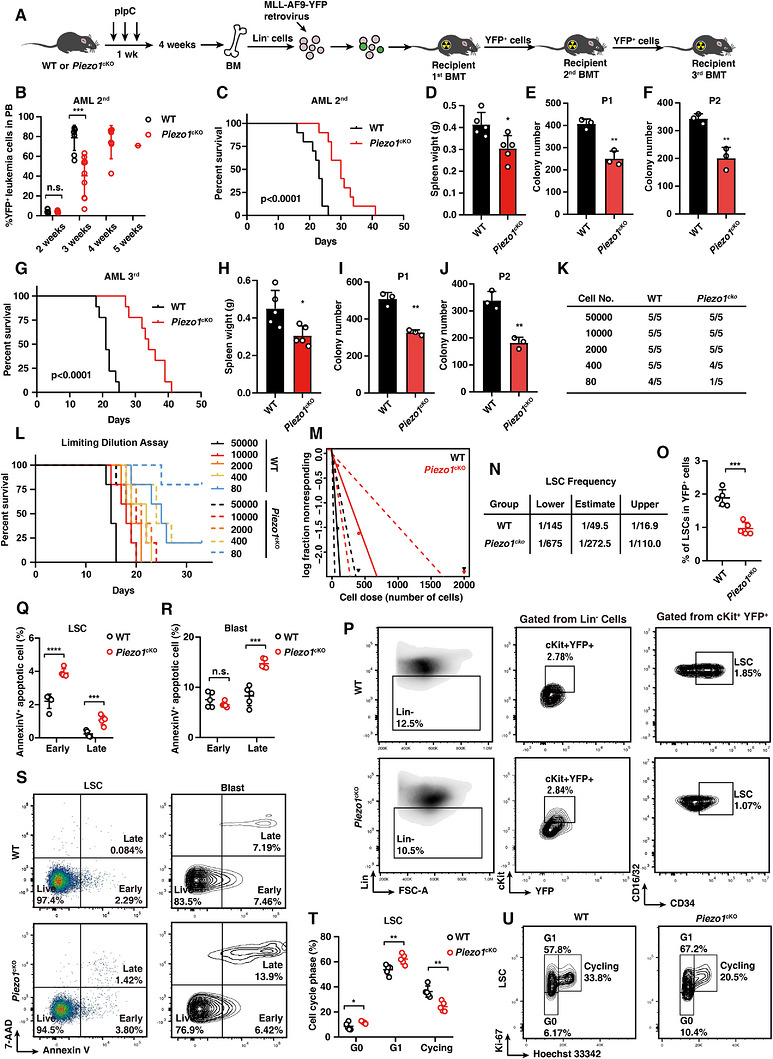
PIEZO1 Is Required for Murine AML Progression. (A) Experimental scheme for (B‐G). (B) Percentages of YFP^+^ leukemia cells in PB from WT or *Piezo1^cKO^* mice at the indicated time after second BMT (*n* = 10 per group). Two‐way ANOVA with Sidak's post‐hoc test. (C) Kaplan‐Meier survival curves for second BMT recipient mice receiving equal number of YFP^+^ leukemia cells from primary AML mice (*n* = 10 per group). Two‐sided log‐rank test. (D) Spleen weight of WT and *Piezo1^cKO^* second BMT recipient mice (*n* = 5). (E) CFU assay of leukemia cells from WT and *Piezo1^cKO^* second BMT recipient mice. (F) CFU assay of colony cells from first plating in (E). (G) Kaplan‐Meier survival curves for third BMT recipient mice receiving equal number of YFP^+^ leukemia cells from second BMT recipient mice (*n* = 10 per group). Two‐sided log‐rank test. (H) Spleen weight of WT and *Piezo1^cKO^* third BMT recipient mice (*n* = 5). Two‐sided log‐rank test. (I) CFU assay of leukemia cells from WT and *Piezo1^cKO^* third BMT recipient mice. (J) CFU assay of colony cells from second plating in (I). (K–N) Limiting dilution assay. Table (K) showing different numbers of cells and recipient mice used in this BMT. Graph (L) showing Kaplan‐Meier survival curves for recipients of limiting dilution assay. Graph (M) and Table (N) showing the ELDA‐calculated frequency of LSCs in WT and *Piezo1^cKO^* AML mice. Statistical significance determined by chi‐square test using ELDA software (http://bioinf.wehi.edu.au/software/elda/). (O) Percentages of LSCs in BM of WT and *Piezo1^cKO^* AML mice (*n* = 5). (P) Representative flow plot showing the percentage of LSCs in total BM of WT and *Piezo1^cKO^* AML mice. (Q,R) Apoptotic analysis of LSCs (Q) and blast cells (R) from WT and *Piezo1^cKO^* AML mice (*n* = 5). (S) Representative flow cytometry plot showing apoptotic analysis of LSCs and blast cells from WT and *Piezo1^cKO^* AML mice. (T) Cell cycle analysis showing percentages of cell cycle phases of LSCs from WT and *Piezo1^cKO^* AML mice (*n* = 5). Two‐tailed unpaired t‐test for each phase. (U) Representative flow cytometry plot showing cell cycle analysis of LSCs from WT and *Piezo1^cKO^* AML mice. *p < 0.05, **p < 0.01, ***p < 0.001 and ****p < 0.0001. Unless otherwise specified in individual panels, all data are presented as mean ± SD (error bars), and statistical significance was determined using two‐tailed unpaired Student's t‐test (paired where indicated). Results are representative of two (B–D, G,H, O–U) and three (E,F, I,J) independent experiments.

To establish an AML mouse model, we transduced Lin^−^ bone marrow (BM) cells derived from WT or *Piezo1^cKO^* donor with the MLL‐AF9‐YFP retrovirus and transplanted them into recipients preconditioned with lethal irradiation (Figure 2A). In the primary transplantation, recipients transplanted with MLL‐AF9‐transduced WT and *Piezo1^cKO^* Lin^−^ cells showed comparable survival and leukemic burden in the PB and spleen (Figure S2J–L). However, we found that Piezo1 deletion markedly impaired AML progression. In the secondary transplantations, AML recipients displayed a decreased leukemic burden in the PB, delayed survival, and reduced the splenic tumor burden (Figure 2B–D; Figure S2M). Colony‐forming unit (CFU) assay also confirmed this phenotype. Leukemia cells sorted from secondary transplantation recipients were used for performing serial colony‐forming unit (CFU) assays, and fewer colonies were observed in the absence of Piezo1 (Figure 2E,F). Similar phenotypes were consistently observed in the tertiary transplantations, as showing the delayed survival, reduced leukemic burden in the spleen, and fewer colonies in the CFU assays (Figure 2G–J; Figure S2N). Thus, these data indicate that Piezo1 deficiency damages LSC function and impairs AML progression. Using a limiting‐dilution assay to quantify LSCs, our analysis revealed a markedly reduced LSC prevalence within the *Piezo1^cKO^* group compared to the WT AML controls (Figure 2K–N). Flow cytometry analysis also confirmed the reduced frequency of LSCs in the *Piezo1^cKO^* samples (Figure 2O,P). Moreover, loss of Piezo1 significantly suppressed entry into the cell cycle and induced apoptosis in LSCs, but only caused a modest increase in apoptosis in blast cells (Figure 2Q–U; Figure S2O,P). In summary, our results reveal that PIEZO1 plays a critical role in sustaining LSC function and murine leukemia progression.

### 2.3 PIEZO1 is Dispensable for Normal Hematopoiesis

**FIGURE 3:**
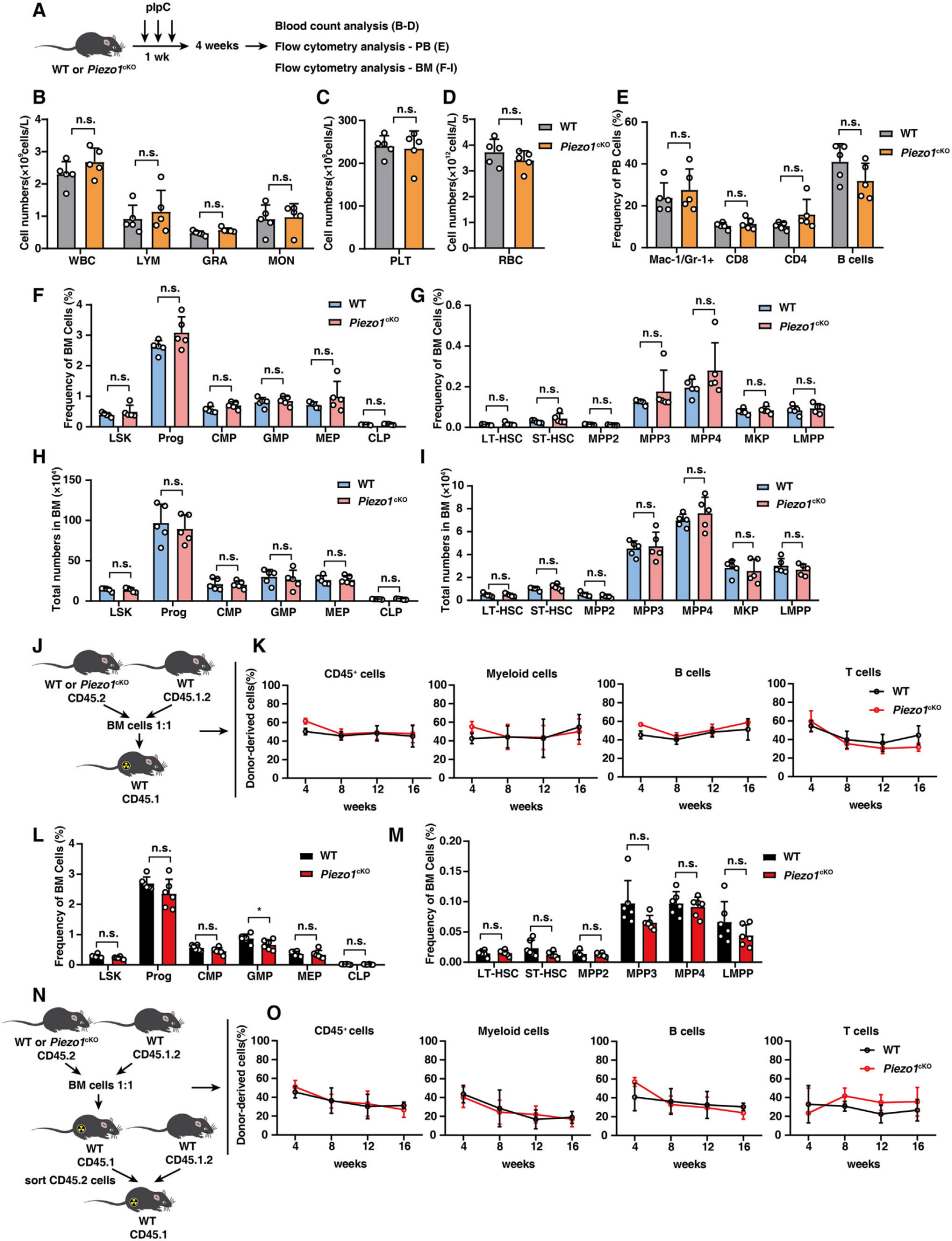
Deletion of PIEZO1 Does Not Affect Murine Normal Hematopoiesis. (A) Experimental scheme for (B–I). (B–D) Blood count analysis in PB of WT and *Piezo1^cKO^* mice (n = 5 per group). (E) Percentages of different lineage cells in PB of WT and *Piezo1^cKO^* mice (*n* = 5 per group). (F,G) Percentages of different stem and progenitor populations in BM of WT and *Piezo1^cKO^* mice (*n* = 5 per group). (H,I) Total numbers of different stem and progenitor populations in BM of WT and *Piezo1^cKO^* mice (*n* = 5 per group). (J) Experimental scheme for (K). (K) Competitive repopulation assay for assessing the reconstitution ability of HSCs. Flow cytometry analysis for different donor‐derived cell lineages in PB of recipient mice at 4, 8, 12, and 16 weeks after BMT (*n* = 5). Two‐way ANOVA with Sidak's post‐hoc test (comparison between WT and cKO at each time point). (L,M) Percentage of donor‐derived progenitor (L) and stem cell (M) compartments in the bone marrow of recipients 16 weeks after BMT. (N) Experimental scheme for (O). (O) Secondary BMT showing comparable long‐term function of WT and *Piezo1^cKO^* HSC. Flow cytometry analysis for different donor‐derived cell lineages in PB of recipient mice at 4, 8, 12, and 16 weeks after BMT (*n* = 5). Two‐way ANOVA with Sidak's post‐hoc test. *p < 0.05, **p < 0.01, ***p < 0.001 and ****p < 0.0001. Unless otherwise specified in individual panels, all data are presented as mean ± SD (error bars), and statistical significance was determined using two‐tailed unpaired Student's t‐test (paired where indicated). Results are representative of two independent experiments (B–I, K–M, O).

We further assessed whether Piezo1 is necessary for normal hematopoiesis. In comparison to WT mice, *Piezo1^cKO^* mice exhibited similar blood counts and proportions of various blood cell populations in the PB, encompassing white blood cells (WBC), lymphocytes (LYM), granulocytes (GRA), monocytes (MON), platelets, red blood cells (RBC), myeloid cells (Mac1^+^, Gr‐1^+^), CD8^+^ T cells, CD4^+^ T cells, B cells (Figure 3A–E; Figure S3A). Furthermore, the deletion of Piezo1 resulted in spleen weights comparable to those of WT mice (Figure S3B,C). Additionally, the proportions and numbers of various hematopoietic stem and progenitor populations in the BM of *Piezo1^cKO^* mice, including LSK, progenitor, CMP, GMP, MEP, CLP, LT‐HSC, ST‐HSC, MPP2, MPP3, MPP4, MKP, and LMPP, showed no statistically significant difference from WT controls. (Figure 3F–I; Figure S3D). Therefore, these results demonstrate that Piezo1 is not essential for normal hematopoiesis.

We subsequently examined the effect of Piezo1 on HSC function. Colony‐forming unit (CFU) assays verified that *Piezo1^cKO^* HSPCs retain normal clonogenic and differentiation potential (Figure S3E,F). To determine HSC function, the competitive repopulation assay was employed (Figure 3J). From 4 to 16 weeks post‐transplantation, the relative frequencies of total donor cells (CD45.2^+^), myeloid cells, B cells (B220^+^), and T cells (CD3e^+^) in the PB of chimeric mice remained comparable across groups. (Figure 3K).

The percentages of donor‑derived progenitor and HSCs in recipient mice showed no statistically significant differences at 16 weeks post‐transplantation (Figure 3L,M). To assess the durability of HSCs, donor‐derived LSK cells were sorted from the recipients at the 16‐week endpoint and subsequently used for secondary transplantation (Figure 3N). From 4 to 12 weeks post‐transplantation, the proportions of donor‐derived cells among various lineages in recipient PB were comparable (Figure 3O). Therefore, these findings demonstrate that PIEZO1 is not essential for normal hematopoiesis and HSC function.

### 2.4 PIEZO1 Deficiency Induces Lipid Peroxidation and Ferroptosis of Leukemia Cells via Impairing Ferroptotic Defense System

**FIGURE 4:**
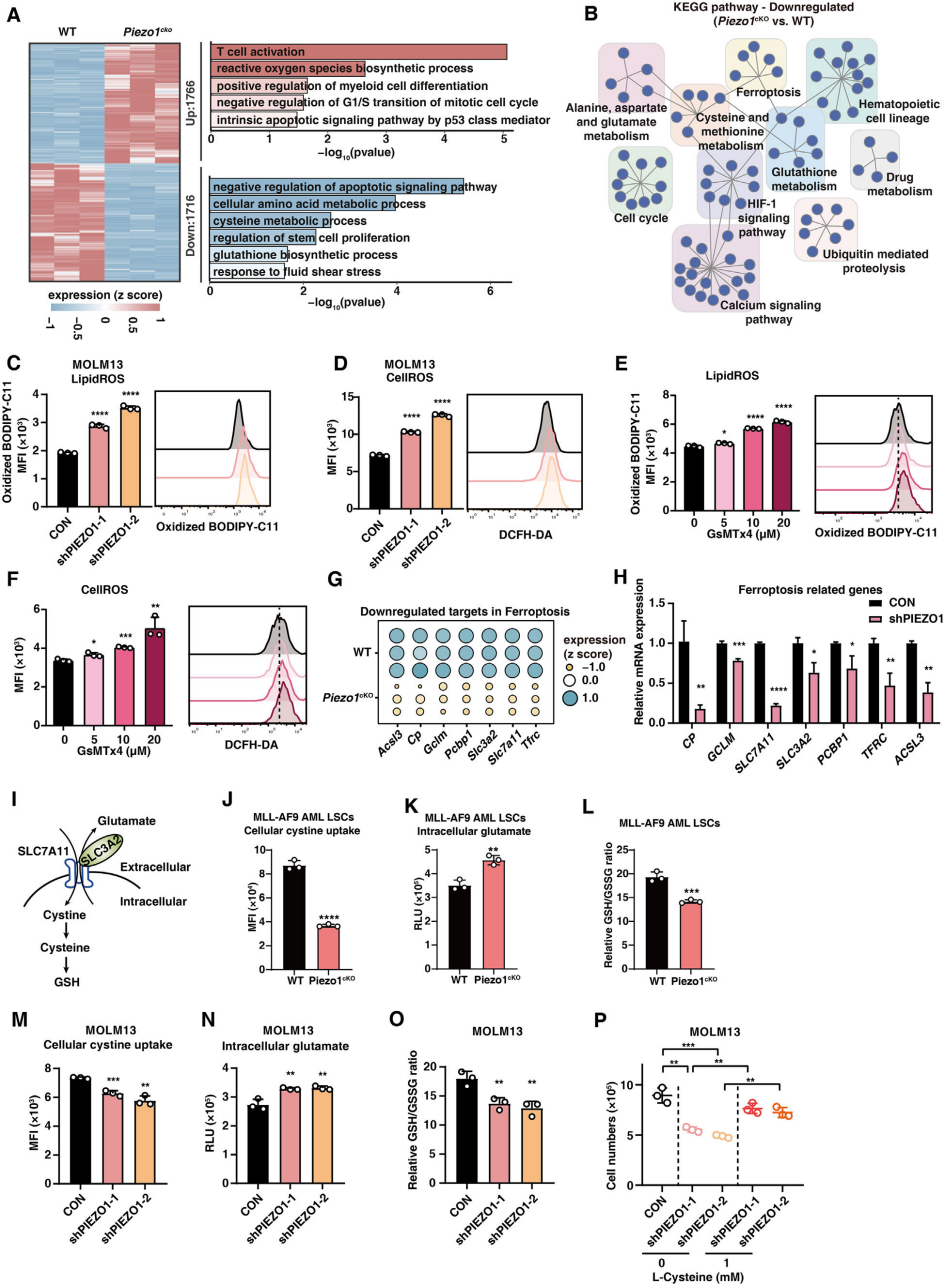
PIEZO1 Protected Leukemia Cells from Ferroptosis in vitro. (A) Heatmap (Left) and GO enrichment analysis (Right) of differential gene expression in WT and *Piezo1^cKO^* LSCs. GO enrichment was performed using Fisher's exact test; adjusted *p* < 0.05 was considered significant. (B) Pathway enrichment analysis for significantly downregulated DEGs in *Piezo1^cKO^* LSCs. Fisher's exact test, adjusted *p* < 0.05. (C,D) Lipid peroxidation levels analysis (C) and ROS levels analysis (D) of MOLM13 leukemia cells after transduction with the indicated lentiviruses. *n* = 3 independent experiments, each in triplicate. One‐way ANOVA with Tukey's post‐hoc test. (E,F) Lipid peroxidation levels analysis (E) and ROS levels analysis (F) of MOLM13 leukemia cells treated with GsMTx4 in different doses (0, 5, 10, 20 µM). *n* = 3 independent experiments, each in triplicate. One‐way ANOVA with Tukey's post‐hoc test. (G) Dot plot showing the downregulated ferroptosis related targets in *Piezo1^cKO^* LSCs. (H) qRT‐PCR showing expression of ferroptosis related genes in MOLM13 leukemia cells. (I) Schematic diagram of amino acid transport through SLC3A2 and SLC7A11. (J–L) Cellular cystine uptake (J), intracellular glutamate level (K) and GSH/GSSG ratio (L) of WT or PIEZO1^cKO^ LSCs from the MLL‐AF9 AML model (*n* = 3). (M–O) Cellular cystine uptake (M), intracellular glutamate level (N) and GSH/GSSG ratio (O) of MOLM13 leukemia cells after transduction with the indicated lentiviruses (*n* = 3). (P) Cell number of MOLM13 leukemia cells after transduction with the indicated lentiviruses in combination with L‐cysteine in different doses (0, 1 mM). *n* = 3 independent experiments, each in triplicate. Two‐way ANOVA with Sidak's post‐hoc test. *p < 0.05, **p < 0.01, ***p < 0.001 and ****p < 0.0001. Unless otherwise specified in individual panels, all data are presented as mean ± SD (error bars), and statistical significance was determined using two‐tailed unpaired Student's t‐test (paired where indicated). Results are representative of three independent experiments (C–F, H, J–M, N–P).

To investigate the role of PIEZO1 in LSC regulation, RNA‐seq was performed on *Piezo1^cKO^* LSCs to assess their gene expression. We identified 3482 DEGs in *Piezo1^cKO^* LSCs, comprising 1766 upregulated and 1716 downregulated (|log2 fold change| >1 and adj‐p‐value <0.05) (Figure 4A). GO analysis showed that the upregulated DEGs were closely associated with T cell activation, reactive oxygen species (ROS) biosynthetic process, myeloid cell differentiation, cell cycle, and apoptosis, while the downregulated DEGs were mainly enriched in cellular amino acid metabolic process, glutathione biosynthetic process, and regulation of stem cell proliferation (Figure 4A). Gene set enrichment analysis (GSEA) further confirmed these findings (Figure S4A–D). Intriguingly, KEGG pathways analysis revealed ferroptotic suppressor genes were enriched in the downregulated DEGs in *Piezo1^cKO^* LSCs (Figure 4B).

It is widely known that ferroptosis is characterized by elevated membrane lipid peroxidation [22]. Quantification of membrane lipid peroxidation with the lipid peroxidation probe BODIPY C11 revealed a significant increase in leukemia cells following PIEZO1 depletion (Figure 4C; Figure S4E). PIEZO1 knockdown also increased the cytosolic ROS level of leukemia cells (Figure 4D; Figure S4F). Similarly, pharmaceutical inhibition of PIEZO1 with either its inhibitor GsMTx4 or Dooku1 also increased the levels of membrane lipid peroxidation and cytosolic ROS in leukemia cells (Figure 4E,F; Figure S4G,H). To distinct ferroptosis from other types of cell death, we treated PIEZO1‐deficient leukemia cells with the inhibitors for ferroptosis, apoptosis, necroptosis, and pyroptosis, respectively. We found that ferroptosis inhibitors Ferrostatin‐1 (Fer‐1) and Liproxstatin‐1 (Lip‐1) markedly reversed PIEZO1‐deficient induced cell death, whereas apoptosis inhibitor Z‐VAD‐FMK, necroptosis inhibitor Nec‐1s, and pyroptosis inhibitor VX765 showed very minimal effects (Figure S4I,J). suggesting that PIEZO1 deficiency mainly causes ferroptosis rather than other types of cell death in AML cells. Together, these findings suggest that PIEZO1 protects leukemia cells from ferroptosis by restraining the accumulation of ROS and lipid peroxidation.

Next, we explored the molecular mechanism by which PIEZO1 protects leukemia cells from ferroptosis. Interestingly, we found that many suppressors of ferroptosis (e.g., *Acsl3, Cp, Gclm, Pcbp1, Slc3a2, Slc7a11*, and *Tfrc*) were markedly downregulated in *Piezo1^cKO^* LSCs (Figure 4G,H). Among these ferroptosis suppressors, SLC7A11 and SLC3A2 form a heterodimer that constitutes the system x_c_^−^ cystine‐glutamate antiporter exchanging extracellular cystine for intracellular glutamate (Figure 4I) [22]. We analyzed cystine uptake and the GSH/GSSG ratio in sorted LSCs from the MLL‐AF9 induced AML model, and found that Piezo1^−/−^ LSCs exhibited reduced cystine uptake but accumulation of intracellular glutamate (Figure 4J,K). The cellular cystine is reduced to cysteine for the biosynthesis of reduced GSH, and the ratio of reduced GSH to oxidized GSH (GSSG) is an indicator of oxidative stress [22]. We also observed a decrease of GSH/GSSG ratio in Piezo1^−/−^ LSCs (Figure 4L). We further measured the cellular cystine and glutamate levels in human leukemia cell line MOLM13. As expected, PIEZO1 knockdown clearly blocked cystine uptake while causing glutamate accumulation (Figure 4M,N). GSH/GSSG ratio was also decreased in leukemia cells with PIEZO1 knockdown (Figure 4O). Finally, supplementation of L‐cysteine partially rescued PIEZO1‐deficient leukemia cells (Figure 4P). Together, our data indicate that PIEZO1 protects AML cells from ferroptosis by regulating SLC7A11/SLC3A2‐mediated cystine uptake.

### 2.5 HIF1A Regulating SLC3A2/SLC7A11 Expression and Mediates the Function of PIEZO1 in AML

**FIGURE 5:**
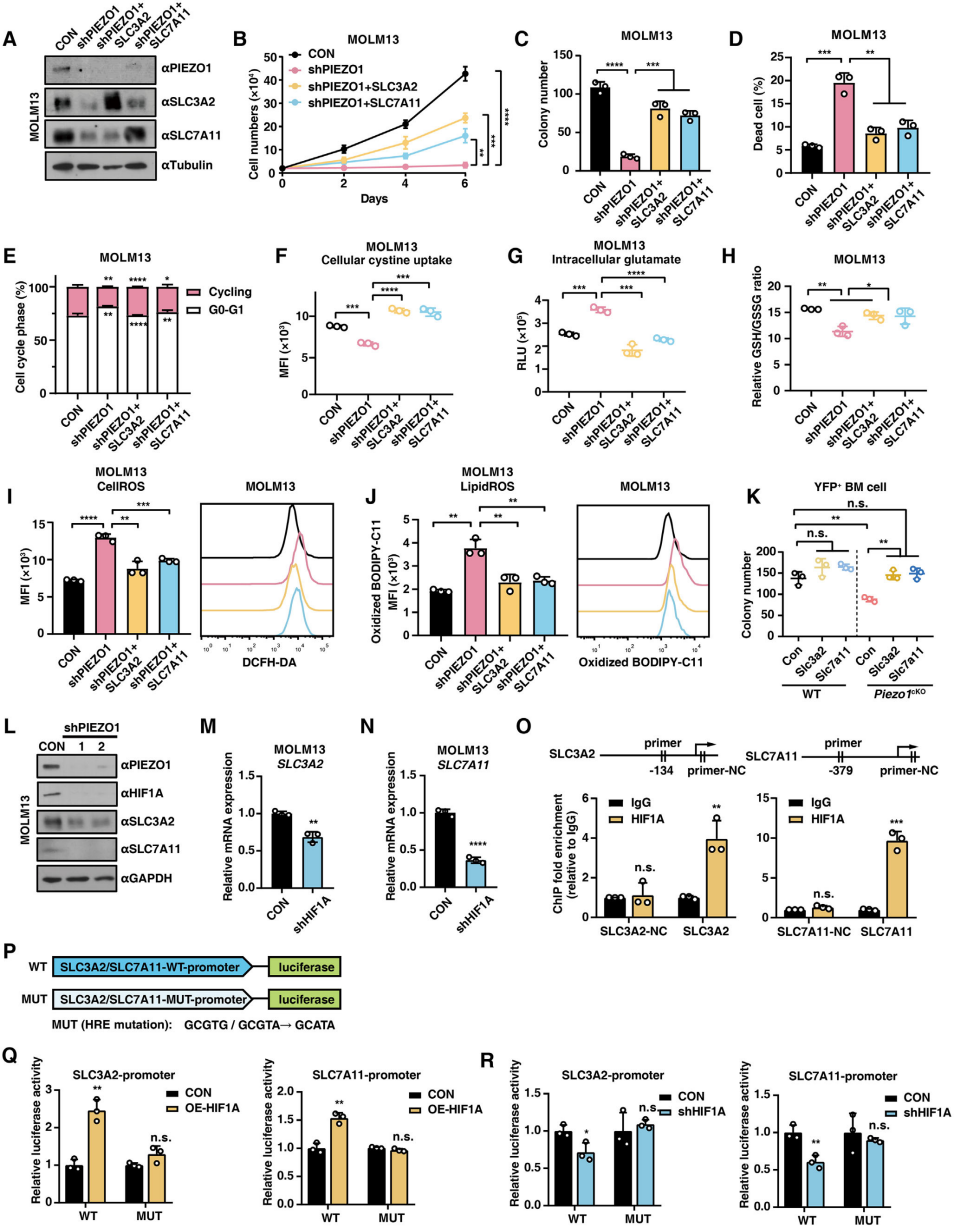
System Xc^−^ Is Required for AML Maintenance. (A) Western blot assay showing expression of PIEZO1, SLC3A2 and SLC7A11 in MOLM13 leukemia cells. Tubulin served as the loading control. (B–J) Growth curves (B), CFU assay (C), cell death analysis (D), cell cycle distribution (E), cellular cystine uptake (F), intracellular glutamate level (G), GSH/GSSG ratio (H), ROS levels analysis (I) and lipid peroxidation levels analysis (J) of MOLM13 leukemia cells after transduction with the indicated lentiviruses. *n* = 3 independent experiments, each in triplicate (except CFU in duplicate). For (B), two‐way ANOVA with Sidak's post‐hoc test. For (C–J), one‐way ANOVA with Tukey's post‐hoc test. (K) CFU assay of YFP^+^ BM leukemia cells from WT and *Piezo1^cKO^* mice after transduction with the indicated lentiviruses. (*n* = 3) Two‐way ANOVA with Tukey's post‐hoc test. (L) Western blot assay showing expression of PIEZO1, HIF1A, SLC3A2 and SLC7A11 in MOLM13 leukemia cells after transduction with the indicated lentiviruses. GAPDH served as the loading control. (M,N) qRT‐PCR showing expression of SLC3A2 (M) and SLC7A11 (N) in MOLM13 leukemia cells after transduction with the indicated lentiviruses (*n* = 3). (O) ChIP‐qPCR assay of HIF1A enrichment in the SLC3A2 and SLC7A11 promoter of MOLM13 leukemia cells. Primers location on SLC3A2 and SLC7A11 promoter (Top) (*n* = 3). (P) Schematic diagram of the structure for WT or MUT luciferase reporter plasmid. (Q,R) Relative luciferase activity of WT or MUT SLC3A2 and SLC7A11 promoter in HEK293T cells transfected with indicated lentiviruses (*n* = 3). *p < 0.05, **p < 0.01, ***p < 0.001 and ****p < 0.0001. Unless otherwise specified in individual panels, all data are presented as mean ± SD (error bars), and statistical significance was determined using two‐tailed unpaired Student's *t*‐test (paired where indicated). Results are representative of two (O) and three (A–N, Q,R) independent experiments.

We next investigated whether SLC3A2 and SLC7A11 mediate PIEZO1 function within AML cells. Ectopic expression of either SLC3A2 or SLC7A11 in PIEZO1‐knockdown leukemia cells partially rescued the defects in proliferation and clonogenicity caused by the absence of PIEZO1 (Figure 5A–C; Figure S5A–D). Similarly, both SLC3A2 and SLC7A11 ameliorated the increased cell death and cell cycling aberrations observed in PIEZO1‐deficient leukemia cells (Figure 5D,E; Figure S5E,F). As expected, ectopic expression of SLC3A2 or SLC7A11 promoted the uptake of cystine, and prevented glutamate accumulation in PIEZO1‐deficient leukemia cells (Figure 5F,G). In addition, both SLC3A2 and SLC7A11 also partially rescued the GSH/GSSG ratio, restored cellular oxidative stress, and membrane lipid peroxidation (Figure 5H–J; Figure S5G,H). Similarly, expression of either Slc3a2 or Slc7a11 also markedly restored the impaired colony‐forming ability of *Piezo1^cKO^* LSCs in the murine AML model (Figure 5K; Figure S5I–K). Thus, our data indicate that SLC3A2 and SLC7A11 mediate the function of PIEZO1 in AML cells.

To investigate how PIEZO1 regulates the expression of SLC3A2 and SLC7A11, we first interrogated their promoter sequences. Intriguingly, the HIF1A binding motifs containing GCGTR were identified in the promoters of both *SLC3A2* and *SLC7A11* (Figure S5L). Additionally, HIF1A protein was significantly decreased upon PIEZO1 deletion (Figure 5L). These data imply that HIF1A might regulate SLC3A2 and SLC7A11 expression. We further knocked down HIF1A in leukemia cells and confirmed that HIF1A deficiency reduced the expression of SLC3A2 and SLC7A11 (Figure 5M,N; Figure S5M). Next, ChIP‐PCR showed the enrichment of HIF1A binding in the promoters of SLC3A2 and SLC7A11 (Figure 5O). To determine whether HIF1A directly regulates SLC3A2 and SLC7A11, we generated firefly luciferase (Fluc) reporter constructs containing either the wild‐type or HIF1A‐binding‐site mutant promoters of both SLC3A2 and SLC7A11 (Figure 5P). Intriguingly, ectopic expression of HIF1A significantly enhanced the luciferase activity of the wild‐type reporter, and such induction was largely blocked by mutation of the HIF1A‐binding‐sites (Figure 5Q; Figure S5N). Similarly, knockdown of HIF1A obviously decreased the Fluc activity of wild‐type reporter, while leaving the mutant promoter construct unaffected (Figure 5R; Figure S5O). Finally, we tested whether HIF1A is a necessary and sufficient downstream mediator of PIEZO1 by overexpressing HIF1A in PIEZO1‐deficient leukemia cells. As expected, HIF1A overexpression substantially reversed the defective phenotypes caused by PIEZO1 deficiency (Figure S5P–T). HIF1A expression also restored cystine uptake, normalized intracellular glutamate accumulation, and partially recovered GSH/GSSG ratio (Figure S5U–W). Both the cellular and lipid ROS levels in PIEZO1‐deficient leukemia cells were also reversed by HIF1A restoration (Figure S5X,Y). Together, these data indicate that the PIEZO1‐HIF1A signaling axis protects AML cells from ferroptosis via SLC3A2/SLC7A11.

### 2.6 Inhibition of PIEZO1‐SLC7A11 Axis Suppresses LSC Function and Contributes to AML Treatment

**FIGURE 6:**
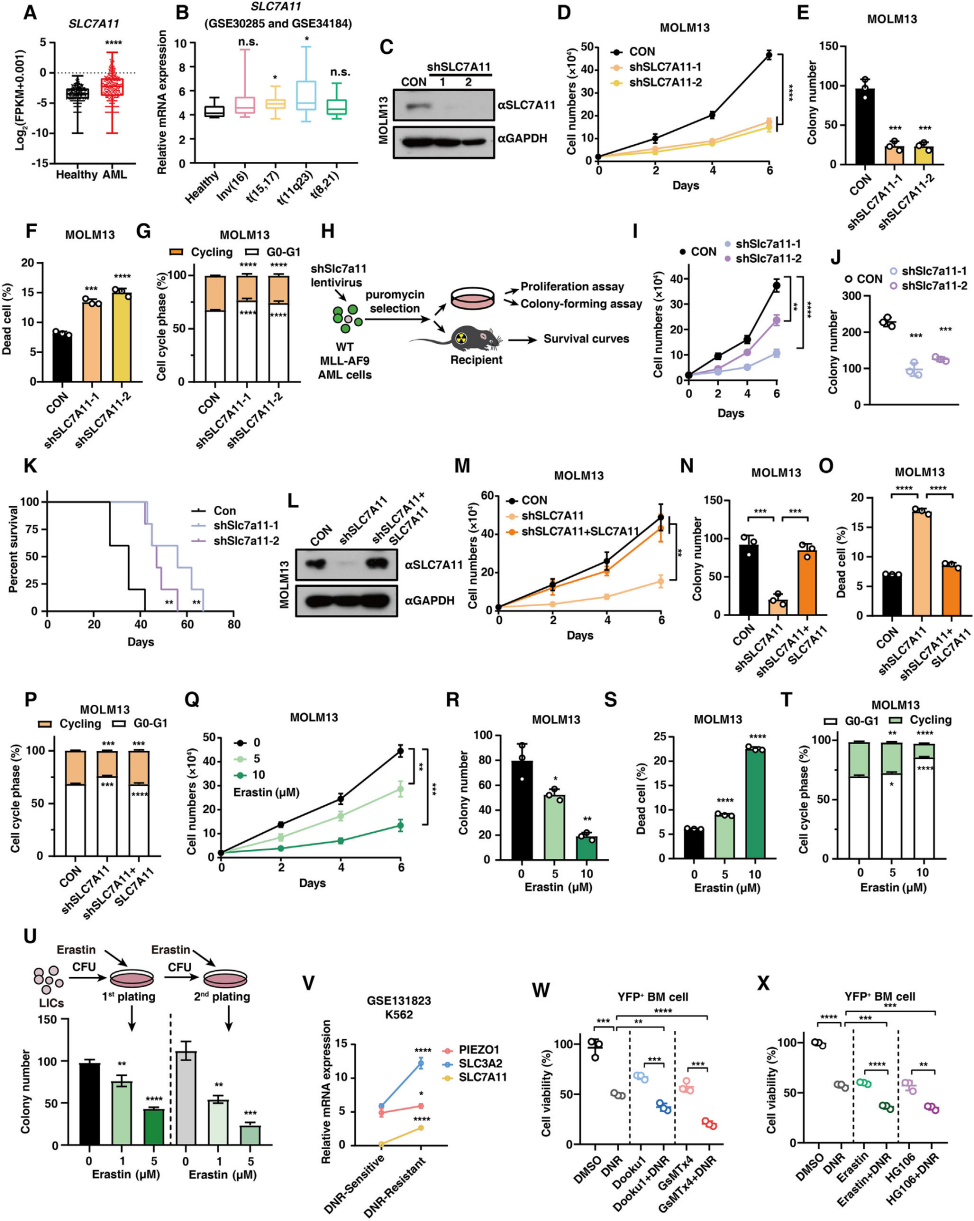
Inhibition of SLC7A11 Suppresses LSC Function and Contributes to Overcoming AML Chemoresistance. (A) Expression of SLC7A11 mRNA in AML patient samples, compared with healthy controls. Data combined from TCGA (*n* = 173), GTEx (*n* = 337), and TARGET (*n* = 196) database. (B) Comparison of SLC7A11 expression in samples of AML patients harboring indicated karyotypes and healthy donors (GSE30285 and GSE34184). (C) Immunoblot showing expression of SLC7A11 in MOLM13 leukemia cells after transduction with the indicated lentiviruses. (D–G) Growth curves (D), CFU assay (E), cell death analysis (F) and cell cycle distribution (G) of MOLM13 leukemia cells after transduction with the indicated lentiviruses. *n* = 3 independent experiments, each in triplicate (except CFU in duplicate). For (D), two‐way ANOVA with Sidak's post‐hoc test. For (E–G), one‐way ANOVA with Tukey's post‐hoc test. (H) Experimental scheme for (I–K). (I,J) Growth curves (I) and CFU assay (J) of mice leukemia cells after transduction with the indicated lentiviruses. n = 3 independent experiments. For (I), two‐way ANOVA with Sidak's post‐hoc test. For (J), one‐way ANOVA with Tukey's post‐hoc test. (K) Kaplan‐Meier survival curves for recipient mice receiving equal number of YFP^+^ leukemia cells after Slc7a11 knockdown (*n* = 5 per group). Two‐sided log‐rank test. (L) Immunoblot showing expression of SLC7A11 in MOLM13 leukemia cells after transduction with the indicated lentiviruses. (M–P) Growth curves (M), CFU assay (N), cell death analysis (O) and cell cycle distribution (P) of MOLM13 leukemia cells after transduction with the indicated lentiviruses. *n* = 3 independent experiments, each in triplicate. For (M), two‐way ANOVA with Sidak's post‐hoc test. For (N–P), one‐way ANOVA with Tukey's post‐hoc test. (Q–T) Growth curves (Q), CFU assay (R), cell death analysis (S) and cell cycle distribution (T) of MOLM13 leukemia cells treated with erastin in different doses (0, 5, 10 µM). *n* = 3 independent experiments, each in triplicate. For (Q), two‐way ANOVA with Dunnett's post‐hoc test. For (R–T), one‐way ANOVA with Dunnett's post‐hoc test. (U) first plating and second plating CFU assay of LICs treated with erastin in different doses (0, 1, 5 µM). *n* = 3 independent experiments. One‐way ANOVA with Dunnett's post‐hoc test. (V) Comparison of PIEZO1, SLC3A2 and SLC7A11 expression in DNR‐sensitive‐K562 leukemia cells and DNR‐resistant‐K562 leukemia cells (GSE131823). (W) Cell viable of YFP^+^ BM cells from WT AML mice treated with DMSO, Dooku1 or GsMTx4 alone, or combining with DNR. (*n* = 3) Two‐way ANOVA with Tukey's post‐hoc test. (X) Cell viability of YFP^+^ BM cells from WT AML mice treated with DMSO, erastin or HG106 alone, or combining with DNR. Two‐way ANOVA with Tukey's post‐hoc test. *p < 0.05, **p < 0.01, ***p < 0.001 and ****p < 0.0001. Unless otherwise specified in individual panels, all data are presented as mean ± SD (error bars), and statistical significance was determined using two‐tailed unpaired Student's t‐test (paired where indicated). Results are representative of two (I–K) and three (C–G, L–U, W,X) independent experiments.

Functioning as one of the major defense mechanisms against ferroptosis, SLC7A11 is critically involved in many tumors [22]. However, the function of SLC7A11 in leukemia has yet to be clearly defined. We observed a higher expression of SLC7A11 in leukemia cells from AML patients (Figure 6A,B). To evaluate its role in AML, we initially reduced SLC7A11 expression in human AML cells using two shRNAs (Figure 6C; Figure S6A–C). The deletion of SLC7A11 significantly suppressed the proliferation and clonogenic ability of leukemia cells, accompanied by increased cell death and cell‐cycle arrest (Figure 6D–G; Figure S6D–G). Additionally, we knocked down Slc7a11 expression in primary murine AML cells (Figure 6H; Figure S6H). As expected, Slc7a11 knockdown significantly decreased proliferation and colony‐forming capacity of leukemia cells (Figure 6I,J). Additionally, we engrafted leukemia cells with Slc7a11 deletion into recipient mice treated with sublethal irradiation, and found that AML progression was significantly delayed in recipients of Slc7a11‐deficient leukemia cells compared to those of the control group (Figure 6K). To exclude potential off‐target effects, we restored SLC7A11 expression in SLC7A11‐deficient leukemia cells (Figure 6L; Figure S6I,J). As expected, enforced expression of SLC7A11 almost completely restored the phenotypical abnormalities of leukemia cells resulting from SLC7A11 deficiency (Figure 6M–P; Figure S6K–N). Thus, these findings suggest that SLC7A11 is indispensable for supporting leukemia cell survival and AML progression.

Furthermore, we attempted to inhibit the activity of SLC7A11 using the selective inhibitor Erastin. As anticipated, treatment with Erastin dose‐dependently suppressed leukemia cell proliferation and colony formation and induced cell death along with cell cycle arrest (Figure 6Q–T). To further evaluate the impact of Erastin on LSC function, we conducted CFU assay using primary murine LICs, and found that treatment with Erastin resulted in a dose‐dependent suppression of LSC clonogenic potential (Figure 6U), suggesting that pharmacological blockade of SLC7A11 impairs AML LSC function.

Daunorubicin (DNR) is the current standard treatment for AML, and resistance to this chemotherapy frequently occurs in clinics. Interestingly, we found that DNR treatment resulted in broad changes in human leukemia cells at the transcriptomic level (Figure S6O). GO analysis showed that the upregulated DEGs associated with DNR resistance were mainly related to lipid metabolic process, amino acid biosynthesis process, and calcium ion transmembrane transport (Figure S6O). Further, we observed obviously higher expression levels of *PIEZO1*, *SLC3A2*, and *SLC7A11* in DNR‐resistant human leukemia cells (Figure 6V). Thus, we evaluated whether combining PIEZO1 inhibitors or SLC7A11 inhibitors with DNR could enhance the eradication of AML cells. Indeed, our research revealed that PIEZO1 inhibitors and SLC7A11 inhibitors act synergistically with DNR to suppress AML cell viability (Figure 6W,X), which provides insight for enhancing future PIEZO1‑targeted therapies for AML. To assess this synergistic effect in vivo, we treated AML mice with GsMTx4 and DNR. As expected, while GsMTx4 or DNR alone modestly delayed disease progression, combination of GsMTx4 and DNR significantly reduced leukemic burden in peripheral blood and delayed AML progression (Figure S6P–R), indicating that pharmacological PIEZO1 inhibition synergizes with DNR to suppress AML progression in vivo. Taken together, our findings reveal that the PIEZO1‐HIF1A‐SLC7A11 axis regulates ferroptosis of AML LSCs.

## 3 Discussion

Exploring AML pathogenesis is urgently needed. Here, we identify the mechanosensor PIEZO1 as a critical regulator of AML, and uncover that LSCs defend oxidative stress and ferroptosis by controlling cystine uptake through the PIEZO1‐HIF1A‐SLC7A11 axis. These findings reveal a unique vulnerability of LSCs.

Our findings demonstrate that the mechano‐sensor PIEZO1 is essential for preserving LSC function and driving AML progression. PIEZO1 is widely expressed, and several studies have focused on its role in different blood cell lineages. For instance, activation of PIEZO1 results in decreased megakaryocyte size, lower ploidy levels, reduced cellular maturation, and diminished proplatelet formation [23]. Its activity also modulates macrophage‐mediated host responses [24, 25]. In addition, myeloid cells are capable of sensing cyclical hydrostatic pressure alterations in their environment via PIEZO1, and of driving a potent and selective proinflammatory response [26]. Gain‐of‐function alterations in PIEZO1, which lead to elevated calcium entry along with enhanced efflux of potassium and water, have been described as being associated with hereditary xerocytosis and myelodysplastic syndromes [27, 28, 29]. However, the specific contribution of PIEZO1 in AML LSCs remains poorly understood. Our work reveals that PIEZO1 is a key factor in promoting AML progression, and uncovers the underlying mechanism by which PIEZO1 maintains LSC stemness.

This study provides a new mechanism for how LSCs tolerate oxidative stress‐induced ferroptosis, which offers a new insight into the vulnerability of LSCs. Although a considerable body of work has focused on ferroptosis execution and regulation [22, 30, 31, 32], deciphering the context‐specific factors governing ferroptosis sensitivity remains a compelling challenge. Mitochondrial reactive oxygen species are central to physiology, and keeping a lower ROS level is critical to protect the stemness of stem cells [33, 34, 35]. Compared to normal HSCs, LSCs experience metabolic reprogramming and display a higher ROS level; thus, understanding how LSCs counteract this oxidative stress is a key for LSC biology. During the onset of ferroptosis, PIEZO1 senses plasma membrane tension and leads to ion fluxes and eventually membrane rupture [36]. Our findings imply that high expression of PIEZO1 increases the potential of LSCs to tolerate excessive oxidative stress and makes them less susceptible to ferroptosis. Mechanistically, PIEZOs are well known as the classical mechanosensors to detect mechanical stress on the plasma membrane, thereby regulating cell fate transitions and maintaining tissue homeostasis [19, 37, 38]. During tumorigenesis and cancer progression, PIEZOs also sense extracellular matrix stiffening‐induced mechanical stimuli from cancer microenvironment, thereby modulating the malignant phenotypes including cancer cells proliferation, migration, invasion, angiogenesis and immune evasion [39]. However, in this study we did not observe significant clue of PIEZO1 mechanosensitive function in AML progression, which may reflect the unique feature of hematologic malignancy. We demonstrate that the function of PIEZO1 in LSCs is mediated by SLC7A11 and SLC3A2, which compose the system x_c_^−^ cystine‐glutamate antiporter. This antiporter serves as a central inhibitor of ferroptosis by mediating cystine uptake. Intracellular cystine is then converted to cysteine, which supports the biosynthesis of glutathione (GSH) via the glutathione (GSH) and/or thioredoxin reductase 1 (TXNRD1) pathway [30, 36]. Interestingly, we observed that exogenous cysteine supplementation only partially rescues PIEZO1‐deficient leukemia cells, implying that reduced expression of SLC7A11 maintains residual activity of this antiporter. Another possibility is that an alternative cystine/cysteine transport system may compensate the compromised function of SLC7A11. However, the precise mechanism of how this antiporter is regulated remains unclear. We found that HIF1A transcriptionally regulates the expression of SLC7A11 and SLC3A2. It should be noted that it remains unclear how PIEZO1 loss downregulates HIF1A expression, which needs to be further investigated. Intriguingly, a recent study found that HSCs are susceptible to ferroptosis due to their low and highly regulated rate of protein synthesis [40]. LSCs have a higher protein synthesis rate than normal HSCs [10], which might provide another avenue for LSCs to defend ferroptosis.

Overall, these findings reveal a new mechanism for LSCs by which LSCs defend against oxidative stress and ferroptosis. This fundamental pathway is critical for LSC maintenance, and beyond that, our findings also have important translational and clinical implications. Furthermore, this study demonstrates that PIEZO1 is not essential for normal hematopoiesis and HSC function, yet its expression correlates with resistance of AML cells to daunorubicin treatment. Therefore, these findings provide a promising therapeutic target for AML, especially for those displaying chemotherapy resistance.

## 4 Materials and Methods

### 4.1 Mice

Piezo1^fl/fl^ mice on the C57BL/6J (CD45.2) background were supplied by Cyagen Biosciences. Mx1‐cre transgenic mice and B6.SJL strain expressing CD45.1 were procured through the Jackson Laboratory. Experimental cohorts included both sexes of mice, aged 8 to 10 weeks, with littermate controls matched for both age and gender. For AML transplantation assays, congenic recipient mice (CD45.2) of the same age range were utilized. All animals were housed and bred within the Animal Center of MRI, Wuhan University. Procedures involving animals were performed in strict adherence to standards authorized by the institution's Animal Care and Use Committee under the approval number WAEF‐2023‐0043.

### 4.2 Primary AML Patient Samples

Bone marrow samples were obtained from AML patients after acquiring informed consent. Ficoll‐based density gradient centrifugation (GE Healthcare Life Science, Cat#17144002) was used to extract Mononuclear cells (MNCs) from the bone marrow. All procedures involving human specimens were performed in accordance with applicable ethical standards and received authorization from the medical research ethics boards of the universities under the approval number WHU‐LFMD‐2023014.

### 4.3 Cell Lines

MOLM‐13, NOMO1 and K562 were maintained in Roswell Park Memorial Institute 1640 medium (Hyclone, RPMI‐1640) containing 10% FBS (Gibco, Cat#10099) and 1% penicillin/streptomycin (HyClone, Cat#SV30010). MV4‐11 was cultured in Iscove's Modified Dulbecco Medium (HyClone, IMDM) with 10% FBS (Gibco, Cat#10099) and 1% penicillin/streptomycin (HyClone, Cat#SV30010). HEK293T were cultured in DMEM (Hyclone), supplemented with 10% FBS (Gibco, Cat#10099) and 1% penicillin/streptomycin (HyClone, Cat#SV30010).

### 4.4 Primary Cell Culture

Lin^−^CD34^+^cells derived from primary AML patients were maintained in StemSpan SFEM (StemCell Technologies) supplemented with rhFLT3L (100 ng mL^−1^, PeproTech, Cat#300‐19), rhIL3 (20 ng mL^−1^, PeproTech, Cat#200‐03), rhTPO (50 ng mL^−1^, PeproTech, Cat#AF‐300‐18‐10), rhSCF (100 ng mL^−1^, PeproTech, Cat#300‐07), 1% penicillin‐streptomycin and 2 mM glutamine (Sigma–Aldrich, Cat#59202C).

### 4.5 Plasmids Constructions

The lentiviral backbones chosen were pLKO.1 and pCDH‐CMV‐Puro in these studies. To silence gene expression, the short hairpin RNAs (shRNAs) of *PIEZO1*, *Piezo1*, *SLC7A11*, *Slc7a1*11 and *HIF1A* were designed and integrated into the pLKO.1 vector. The complete list of shRNA target sequences was catalogued in the Table S1. For rescue assays, cDNA encoding human SLC3A2 or SLC7A11 was inserted into vector following shPIEZO1 or shSLC7A11. To achieve the overexpression of *Slc3a2*, *Slc7a11*, and *HIF1A*, the pCDH‐CMV‐Puro lentiviral vector was employed via routine molecular cloning procedures. SLC3A2 and SLC7A11 promoter were amplified by PCR with targeted primers and subsequently ligated into pGL3‐basic luciferase reporter plasmid through designated restriction sites. The HIF1A HRE binding sites in pGL3‐SLC3A2‐promoter‐MUT or pGL3‐SLC7A11‐promoter‐MUT were mutated from GCGTG or GCGTA into GCATA using specific primers.

### 4.6 Lentivirus Production and Transduction

To generate lentiviral particles, HEK293T cells were subjected to co‐transfection with the packaging vectors pSPAX2 and pMD2.G, employing polyethyleneimine (PEI, Polysciences, Cat#24765‐1) as the transfection reagent. Retrovirus were generated via the transfection of HEK293T cells with the packaging plasmid pCL‐eco, using calcium phosphate precipitation method. Viral supernatants were gathered and filtered through a 0.45 µm disposable syringe filter (Millipore, Cat#SLHV033RB) at 48‐ and 72 h post‐transfection. Target cells were exposed to the virus in medium containing 8 µg mL^−1^ polybrene (Sigma–Aldrich, Cat#H9268), accompanied by centrifugation at 2200 rpm. The culture medium was replaced 12 h post‐infection, and when selection was required, puromycin (1‐2 µg mL^−1^) were supplemented 2 days post‐infection.

### 4.7 Generation of the Murine MLL‐AF9 Leukemia Model

Bone marrow cells were isolated from Piezo1^cKO^ and WT mice aged 8–10 weeks, and Lin^−^ cells (lineage‐negative) were purified using the hematopoietic stem/progenitor cell enrichment kit (StemCell, Cat#19856A). These purified cells were then subjected to two rounds of infection with MSCV‐MLL‐AF9‐IRES‐YFP retrovirus in medium containing SCF (20 ng mL^−1^, PeproTech, Cat#250‐03), IL‐6 (10 ng mL^−1^, PeproTech, Cat#216‐16), IL‐3 (10 ng mL^−1^, PeproTech, Cat#213‐13) and 8 µg mL^−1^ polybrene. Subsequently, infected cells (2 × 10^5^ – 3 × 10^5^) were injected intravenously into recipient C57BL/6 mice that had received sub‐lethally (4 Gy + 4 Gy) irradiation. For secondary and tertiary transplantation, 3000 YFP^+^ leukemia cells isolated from bone marrow of primary diseased mice was employed. Peripheral blood was collected and assessed weekly to measure the percentage of YFP^+^ leukemia cells.

In the limiting dilution assay, graded doses of 5 × 10^4^, 1 × 10^4^, 2 × 10^3^, 4 × 10^2^, and 80 YFP^+^ leukemia cells were injected intravenously into recipient C57BL/6 mice that had received sub‐lethally (4 Gy+4 Gy) irradiation. The frequency of LSCs was determined through statistical analysis using the ELDA software.

### 4.8 Flow Cytometry Analysis and Cell Sorting

Bone marrow cells from mice were treated with an erythrocyte lysis solution on ice for five minutes to remove erythrocytes, followed by washing with PBS containing 2% FBS. The processed cells were subjected to cell surface marker staining. For mouse HSPC and LSC studies, cell surface markers are provided in the supplemental Data.

To examine mature cell populations, PB cells were marked with Anti‐B220‐PE‐Cy5, Anti‐CD8‐FITC, Anti‐CD4‐APC, Anti‐Gr‐1‐PE‐Cy7, Anti‐CD11b‐PE. In the competitive repopulation experiment, PB samples were first incubated with Anti CD45.1‐BV421 and Anti‐CD45.2‐PE‐CF594 for the identification of CD45.2^+^ donor‐derived cells, and then further stained with Anti CD3e‐FITC, Anti B220‐APC‐Cy7, Anti Gr‐1‐APC, and Anti CD11b‐PE.

For mouse stem/progenitor cell isolation, the EasySep Mouse Hematopoietic Progenitor Cell Isolation Kit (StemCell, Cat#19856) was utilized to enrich Lineage‐negative progenitors from bone marrow following the provided protocol. After washing, cells were incubated with the anti‐Streptavidin to recognize biotin, then labeled with Anti‐CD34‐AF700, Anti‐CD16/32‐PE‐Cy7, Anti‐c‐Kit‐PE, Anti‐Sca‐1‐APC. Viability was assessed through co‐staining with 7‐AAD. Subsequently, LSCs were obtained by flow cytometer sorting using a BD FACS Aria III instrument. Human CD34^+^ leukemia stem/progenitor cells were positively selected with CD34‐conjugated microbeads using a magnetic separation system (Miltenyi, Cat#130‐046‐702).

BD LSRFortessaX‐20, FACSCelesta, Beckman Cytoflex LX and Cytoflex were employed for cell phenotypic analysis, and BD FACS Aria III was utilized for cell sorting at the Flow Cytometry Core Facility of MRI. The resulting datasets were processed with FlowJo.

### 4.9 Cell Proliferation and Colony‐Forming Assay

For leukemia cell proliferation assays, cells were inoculated into 24‐well plates at 2 × 10^4^ cells per well, with three replicate wells per group. Cell counting was performed at two‐day intervals. For the colony‐forming assays, lentivirus‐transduced CD34^+^ cells from AML patients were seeded in MethoCult H4434 methylcellulose medium (StemCell Technologies) following the provided protocol, while mouse bone marrow cells were grown in MethoCult M3434 methylcellulose medium (StemCell Technologies). For cell lines, transduced cells were inoculated in a semi‐solid medium of 1.2% methylcellulose containing 10% FBS and 1% penicillin/streptomycin, with three replicates per group. Colony formation was assessed and quantified following 5–12 days of incubation.

### 4.10 Cell Cycle and Apoptosis Analysis

Cell cycle profiling of leukemia cells was carried out by incubating the cells with Hoechst 33342 (ThermoFisher, Cat#H3570) at 37°C for 30 min, and then examined by flow cytometric. Primary mouse BM cell cycle analysis was assessed by incubating cells with Hoechst 33342 at 37°C for 90 min, followed by surface marker labeling and intracellular Ki‐67 staining. Cells were subjected to flow cytometric analysis, and the resulting datasets were evaluated using FlowJo. For apoptosis analysis, human leukemia cells were labeled with Annexin V (BD Biosciences, Cat#556547) and PI prior to flow cytometry. Mouse BM cells were similarly processed using Annexin V (BD Biosciences, Cat#556547), followed by the addition of 7‐AAD (StemCell, Cat#75001) before flow cytometric analysis.

### 4.11 Cell Viability, Death, and Death Pathway Assay

The Cell Counting Kit‐8 (CCK‐8, Biosharp, BS350B) was used to assess cell viability. Mouse BM cells were inoculated into 96‐well plates at a density of 5 × 10^4^ cells per well in triplicates, and treated with indicated conditions or compounds. After 36 h of culture, 10 µL of CCK‐8 solution was dispensed into every well. After 2–4 h reaction, the absorbance at 450 nm was detected on the microplate reader. Cell viability was calculated as:

$$\mathrm{Viab}\mathrm{ility}\;\left(\%\right)=\left(OD_{\mathrm{trea}\mathrm{ted}}-OD_{\mathrm{blank}}\right)/\left(OD_{\mathrm{cont}\mathrm{rol}}-OD_{\mathrm{blank}}\right)\times 100$$

Cell death was estimated as 100% − viability (%). All experiments were performed in triplicate.

To identify the type of cell death, suspension cells were virus‐infected, selected with puromycin for 2 days, and treated for 36 h with cell death inhibitors: Ferrostatin‐1 (5 µM, MCE, Cat#HY100579) and Liproxstatin‐1 (5 µM, MCE, Cat#HY‐12726) for ferroptosis, Z‐VAD‐FMK (20 µM, MCE, Cat#HY‐16658B) for apoptosis, Necrostatin‐1 (10 µM, Nec‐1s, MCE, Cat#HY‐15760) for necroptosis, and Belnacasan (VX‐765) (20 µM, Selleckchem, Cat#S2228) for pyroptosis. Cell viability was measured using CCK‐8, with three replicates per condition. Rescue of viability by each inhibitor was used to identify the corresponding cell death pathway.

### 4.12 Competitive Repopulation Assay

A total of 5 × 10^5^ donor bone marrow cells isolated from Piezo1^cKO^ or WT mice were combined with an equivalent number of competitors CD45.1.2 cells and co‐transplanted into CD45.1 recipients that had received lethal split‐dose irradiation (5 Gy + 5 Gy). Subsequently, the repopulation ability and multiple lineages of donor cells were analyzed at 4‐, 8‐, 12‐, and 16‐weeks post‐transplantation. At the 16‐week time point, donor‐derived bone marrow cells were subjected to FACS analysis, after which cells from the primary recipients were combined with an equivalent number of competitor cells and transferred into secondary CD45.1 mice that had undergone lethal split‐dose irradiation (5 Gy + 5 Gy).

### 4.13 Reactive Oxygen Species (ROS) Measurement

Reactive oxygen levels were measured by staining cells with DCFH‐DA (10 µM, Beyotime, Cat# S0033S) for 20 min at 37°C. Afterward, the cells were washed three times with serum‐free medium and allowed to incubate for an additional hour at 37°C before being analyzed by flow cytometry.

### 4.14 Lipid Peroxidation Measurement

Lipid peroxidation levels were assessed by staining cells with BODIPY 581/591 C11 reagent (5 µM, Invitrogen, Cat# D3861) and incubating them at 37°C for 30 min. ROS levels were subsequently determined via flow cytometry.

### 4.15 Cystine Measurement

Intracellular cystine levels were assessed by staining cells with BioTracker Cystine‐FITC Live Cell Dye (Merck, Cat# SCT047, 5 µM) for 20–40 min at 37°C. After incubation and washing, the cells were assessed via flow cytometry.

### 4.16 Glutamate Measurement

Intracellular glutamate levels were quantified using the Glutamate‐Glo Assay (Promega, Cat# J7021). The experiment was performed in 96‐well plates, in which 2 × 10^4^ cells were plated per well, rinsed twice with PBS, and then resuspended in 25 µL of PBS. Cell lysis was initiated by the addition of 12.5 µL per well of Inactivation Solution (0.6 N HCl), followed by a 5 min incubation. Subsequently, an equal volume of Neutralization Solution was added to neutralize the reaction. After addition of 50 µL/well Glutamate Detection Reagent, the plates were incubated for 1 h at room temperature. Luminescence intensity was detected with a SpectraMax iD3 microplate reader (Molecular Devices).

### 4.17 Measurement of Intracellular GSH/GSSG Ratio

The levels of total and oxidized glutathione were quantified with the GSH/GSSG‐Glo Assay Kit (Promega, Cat# V6611). The experiment was performed in white 96‐well plates at room temperature. Briefly, cells were plated at a density of 5× 10^4^ cells per well, washed twice with HBSS (Hank's Balanced Salt Solution), and then resuspended in 25 µL of HBSS. After adding 25 µL of respective glutathione lysis reagent (total or oxidized) to each well, the plate was agitated for 5 min to lyse cells. Then, 50 µL of the Luciferin Generation Reagent was added to the lysate, followed by a 30 min incubation. Finally, 100 µL Luciferin Detection Reagent was introduced per well, and the mixture was reacted for 15 min. Luminescence intensity was finally detected with a SpectraMax iD3 microplate reader (Molecular Devices).

The GSH/GSSG ratio was calculated using as:

$$\begin{array}{ccc} \mathrm{GSH}/\mathrm{GSSG} & = & \left(\mathrm{total}\;\;\;\mathrm{glutathione}\;\;\mathrm{RLU}-\mathrm{GSSG}\;\;\mathrm{RLU}\right)\;/\;\\ & & \left[\mathrm{GSSG}\;\;\mathrm{RLU}/2\right]. \end{array}$$

### 4.18 Western Blot

Cells were subjected to RIPA lysis buffer containing protease inhibitors cocktail (Roche, Cat#11873580001). Then, the protein lysates were separated by SDS‐PAGE and transferred onto a NC membrane via wet transfer. After blocking with 5% non‐fat milk in TBS (Tris‐buffered saline) for 30 min, the membrane was incubated with primary antibodies overnight at 4°C. The primary antibodies and corresponding dilution ratios applied were as follows: Anti‐PIEZO1 (HUABIO, Cat#M1005‐2, 1:500), Anti‐SLC3A2 (Proteintech, Cat#15193‐1‐AP, 1:1000), Anti‐SLC7A11 (Cell Signaling Technology, Cat#12691, 1:1000), Anti‐ Tubulin (Abcam, Cat#ab6160, 1:2000), Anti‐GAPDH (Proteintech, Cat#60004, 1:2000) and Anti‐HIF1A (proteintech, Cat#20960‐1‐AP, 1:1000). On the following day, the membrane was exposed to secondary antibodies for 1 h at room temperature after washing with TBST (0.1% Tween‐20 in TBS). Then, the protein bands were detected using ECL Western Blotting Substrate (Bio‐Rad, Cat#1705061) and visualized by exposure to X‐ray film (Super RX, Fujifilm).

### 4.19 RNA Isolation, cDNA Synthesis and qRT–PCR

Total RNA was extracted with TRIzol reagent (Takara). The Toyobo's ReverTra Ace qPCR RT Kit (FSQ‐101) was utilized to carry out reverse transcription. Next, Quantitative real‐time PCR analysis was conducted on a CFX384 Real‐Time PCR System (BioRad) with the Power SYBR Green PCR Master Mix (BioRad, Cat #AA.1725124). Target gene mRNA expression levels was normalized to the housekeeping gene Gapdh.

### 4.20 ChIP‐PCR

For ChIP‐PCR, 3 × 10^6^ MOLM13 cells were crosslinked in 1% formaldehyde solution for 10 min at room temperature. The crosslinking was terminated by adding 0.125 M glycine, followed by a 5 min incubation. Then cells were lysed on ice for 10 min by resuspension in a Hypotonic Lysis buffer. After washing with a buffer containing 1 mM EDTA and 10 mM Tris‐HCl (pH 7.9), the chromatin was fragmented in a shearing buffer (containing 0.1% SDS in addition to the above components) using a Bioruptor sonicator. Immunoprecipitation was performed using either 2 µg of anti‐HIF1A (proteintech, Cat#20960‐1‐AP) or control IgG antibodies (proteintech, Cat#30000‐0‐AP). The immunoprecipitated complexes underwent multiple rounds of washing in sequence: twice with IP buffer, twice with High‐salt IP buffer, twice with 27 LiCl buffer, and finally twice with TE buffer. The chromatin captured by immunoprecipitation was eluted and crosslinking was reversed by overnight incubation at 65°C, followed by proteinase K (Thermo Fisher, Cat#25530049) and RNase A (Thermo Fisher, Cat#EN0531) treatments. Eluted chromatin was purified using phenol‐chloroform, precipitated with ethanol, and dissolved in H2O. DNA Samples were analyzed by qPCR. 10% of the fragmented chromatin was directly extracted prior to immunoprecipitation and amplified by PCR using the identical primers as input control. Relevant reagent formulations are listed in Supplemental Data.

### 4.21 Luciferase Reporter Assay

HEK293T cells were planted in 24‐well plates and subsequently transfected with the pGL3‐REPORT luciferase reporter construct fused with WT or HIF1A HRE binding sites mutant SLC3A2/SLC7A11 promoter. The pRL‐TK Renilla luciferase plasmid (Promega) was co‐delivered into the cells as an internal reference standard for normalization. Cells were collected 24 h after HIF1A overexpression or 48 h after shHIF1A treatment. The Dual‐Luciferase Reporter Assay System (Promega, Cat# 1980) was employed to assess the activities of Firefly and Renilla luciferase, following the provided instructions. Relative luciferase activity was measured by calculating the ratio of firefly to Renilla signals and normalized to the control group under the same experimental conditions.

### 4.22 RNA‐Seq and Data Analysis

Total RNA extraction was achieved with TRIzol reagent (Takara). Construction of the RNA‐seq library followed our tailored protocol derived from Smart‐seq2, as previously reported. In brief, total RNA underwent reverse transcription using a VN‐anchored oligo‐dT primer alongside a template‐switching oligonucleotide (TSO), followed by pre‐amplification of the full‐length cDNA using the ISPCR primer. The pre‐amplified cDNA was subjected to fragmentation via Tn5 transposase. Subsequently, the resulting fragments were then processed into sequencing libraries by means of the Vazyme TruePrep DNA Library Prep Kit V2 for Illumina (Product Code: TD501). Finally, Illumina HiSeq X Ten platform was used to perform paired‐end sequencing on these produced libraries.

For sequencing data derived from WT and Piezo1^cKO^ LSCs, initial processing of the raw reads was performed with fastp (V0.20.1). The clean reads subsequently were mapped to the GRCm38 transcripts database using hisat2 (V2.2.1), with subsequent gene expression quantification performed via featureCounts (V2.0.1). The DESeq2 package (version 1.28.1) was applied to normalize the RNA‐seq count data and to perform statistical testing for differential expression between WT and Piezo1^cKO^ LSCs. Differentially expressed genes (DEGs) were filtered by setting the criteria of |log2 fold change| > 1 and adj‐p‐value < 0.05. Gene Ontology (GO) functional enrichment analysis was conducted utilizing Cytoscape (V 3.7.2) in conjunction with the clusterProfiler package (V3.16.0). Results from the Gene Set Enrichment Analysis (GSEA) were graphically represented by the GSEA software (version 4.1.0).

### 4.23 Statistical Analysis

Statistical analysis of experimental data was executed with GraphPad Prism (version 9). No data transformation or normalization was applied. Outliers were not excluded; all raw data were retained. Data are presented as mean ± SD (error bars). Two‐tailed Student's *t*‐test was employed to evaluate the intergroup variations. To assess differences between distinct samples, the unpaired t‐test was applied. To determine differences between treatments applied to the same sample, the paired t‐test was employed. For multiple comparisons, one‐way ANOVA with Tukey‘s post‐hoc test was applied. Survival curve comparisons were carried out using the log‐rank test. *P* < 0.05 was deemed statistically significant, while *p* > 0.05 was categorized as non‐significant (n.s.). Statistical significance was defined as follows: *p* < 0.05 (*), *p* < 0.01 (**), *p* < 0.001 (***), *p* < 0.0001 (****). Sample sizes (n) are provided in each figure legend. The experiments were repeatedly verified multiple times, among them, the animal assays were replicated at least twice, and the cell experiments were performed with at least three independent repetitions.

## Author Contributions

**T.Z**., and **H.Z**. conceived the project, designed experiments, analyzed the data, and wrote the manuscript; **T.Z**., **Z.C**. and **M.C**. performed in vitro function and mechanisms studies with the help of **J.W**. and **R.Y**.; **T.Z**. performed bioinformatic analyses; **T.Z**., **Z.C**., and **M.C**. performed mouse experiments with the help of **J.W**., **W.L**., and **G.H**.; **C.W**. provided primary patient samples; **C.W**. and **H.Z**. supervised the overall study.

## Consent

No written content for publication has been obtained from the patients as there is no patient identifiable data included in this article.

## Conflicts of Interest

The authors declare no conflicts of interest.

## Supporting information




**Supporting File**: advs75648‐sup‐0001‐SuppMat.pdf.

## Data Availability

The accession numbers for the RNA‐seq data presented in this study have been deposited in public database. The GSE number is GSE313852.
