# Supplementary material for: PIEZO1 is Required for Acute Myeloid Leukemia Progression and Leukemia Stem Cell Maintenance via HIF1A‐SLC7A11 Axis‐Mediated Ferroptosis Defense
Source: Adv Sci (Weinh). 2026 May 10;13(44):e75648. doi: 10.1002/advs.75648 (PMC13335963; doi:10.1002/advs.75648)
Supplement: Supplementary file 1 [file ADVS-13-e75648-s001.pdf]

Supplementary Information

PIEZO1 is required for acute myeloid leukemia progression and leukemia stem cell maintenance via HIF1A-SLC7A11 axis-mediated ferroptosis defense

Tiantian Zhang[1,2,6], Ziyan Cao[1,2,6], Kexin Gao[2,6], Shuxin Yao[2], Yanbing Zheng[2], Manman Cui[2], Rong Yin[2,3], Guoqiang Han[1,2,3], Jin Hu[2*], Chengyi Wang[4*], Haojian Zhang[1,2,5*]

[1]State Key Laboratory of Oral & Maxillofacial Reconstruction and Regeneration, Key Laboratory of Oral Biomedicine Ministry of Education, Hubei Key Laboratory of Stomatology, School & Hospital of Stomatology, Wuhan University, Wuhan, China;
[2]Frontier Science Center for Immunology and Metabolism, Medical Research Institute, Wuhan University, Wuhan, China;
[3]Department of Hematology, Zhongnan Hospital, Wuhan University, Wuhan, China;
[4]Department of Pediatrics, Fujian Children's Hospital (Fujian Branch of Shanghai Children's Medical Center), College of Clinical Medicine for Obstetrics & Gynecology and Pediatrics, Fujian Medical University
[5]Taikang Center for Life and Medical Sciences, Wuhan University, Wuhan, China;
[6]These authors contributed equally;

*Correspondence Email: haojian_zhang@whu.edu.cn; wangchengyi79@126.com; hujin1119@whu.edu.cn

Supplementary Figures

Fig. S1 Elevated expression of PIEZO1 Is Required for Survival of Human Myeloid Leukemia Cells
Fig. S2 Piezo1 Is Required for Murine AML Development
Fig. S3 Deletion of Piezo1 Does Not Affect Murine Normal Hematopoiesis
Fig. S4 PIEZO1 Protected Leukemia Cells from Ferroptosis in vitro
Fig. S5 System Xc⁻ Is Required for AML Maintenance
Fig. S6 Inhibition of PIEZO1-SLC7A11 Axis Suppresses LSC Function and Contributes to Overcoming AML Chemoresistance

Table S1 List of Primer sequences

Supplementary Materials and Methods

Supplementary Figure 1

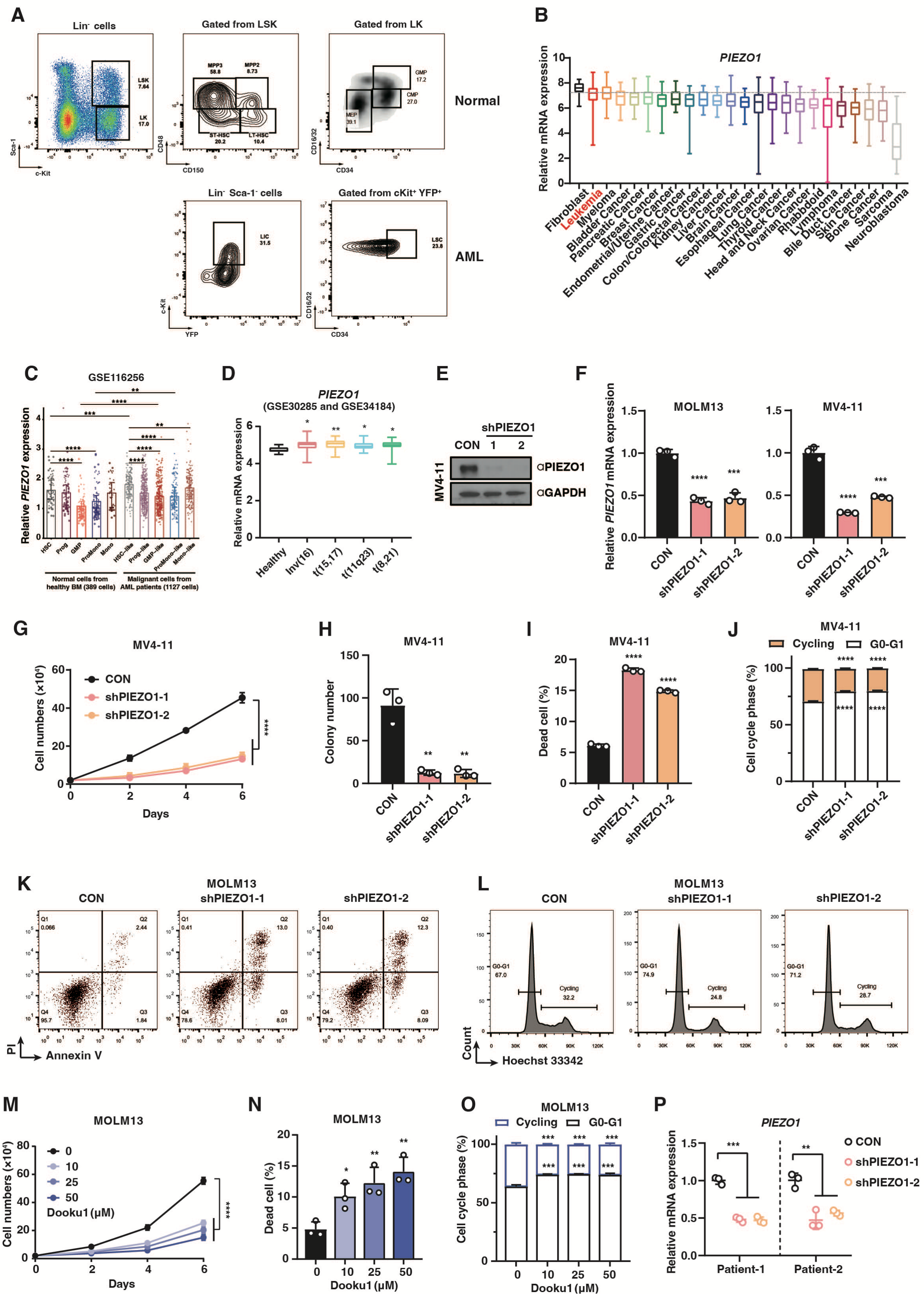

Fig. S1 Elevated expression of PIEZO1 Is Required for Survival of Human Myeloid Leukemia Cells

(A) The representative gating strategy for flow cytometry analysis and cell sorting of different bone marrow cell populations from murine AML mice and healthy control mice. This gating strategy is used for Figure 1A and 2O.

(B) PIEZO1 mRNA level in AML or other cancers among 1429 human cancer cell lines in the Can Cell Line Encyclopedia database.

(C) Single-cell RNA-seq data showing *PIEZO1* expression in healthy donor- and AML patient-derived bone marrow cells. Each dot represents one cell (GSE116256).

(D) Comparison of PIEZO1 expression in samples of AML patients harboring indicated karyotypes and healthy donors (GSE30285 and GSE34184).

(E) Western blot assay showing knockdown efficiency of PIEZO1 in MV4-11 leukemia cells. GAPDH served as the loading control.

(F) qRT-PCR showing knockdown efficiency of PIEZO1 in MOLM13 (Left) and MV4-11 (Right) leukemia cells. n=3, independent experiments.

(G-J) Growth curves **(G)**, CFU assay **(H)**, cell death analysis **(I)** and cell cycle distribution **(J)** of MV4-11 leukemia cells after transduction with the indicated lentiviruses. n=3 independent experiments, each in triplicate (except CFU in duplicate). For (G), two-way ANOVA with Sidak's post-hoc test. For (H-J), one-way ANOVA with Tukey's post-hoc test.

(K-L) Representative flow cytometry plot showing apoptotic **(K)** and cell cycle **(L)** analysis of MOLM13 leukemia cells after transduction with the indicated lentiviruses.

(M-O) Growth curves **(M)**, cell death analysis **(N)** and cell cycle distribution **(O)** of MOLM13 leukemia cells treated with Dooku1 in different doses (0, 10, 25, 50 μM). n=3, independent experiments, each in triplicate. For (K), two-way ANOVA with Dunnett's post-hoc test. For (L-M), one-way ANOVA with Dunnett's post-hoc test.

(P) qRT-PCR showing knockdown efficiency of PIEZO1 in AML patient-derived $Lin^{-}CD34^{+}$ cells. n=2, independent patient samples (each measured in triplicate)

*p < 0.05, **p < 0.01, ***p < 0.001 and ****p < 0.0001. Unless otherwise specified in individual panels, all data are presented as mean ± SD (error bars), and statistical significance was determined using two-tailed unpaired Student's t-test (paired where indicated). Error bars denote mean ± SD. Results are representative of two (**P**) and three independent experiments (**E-O**).

Supplemental Figure 2

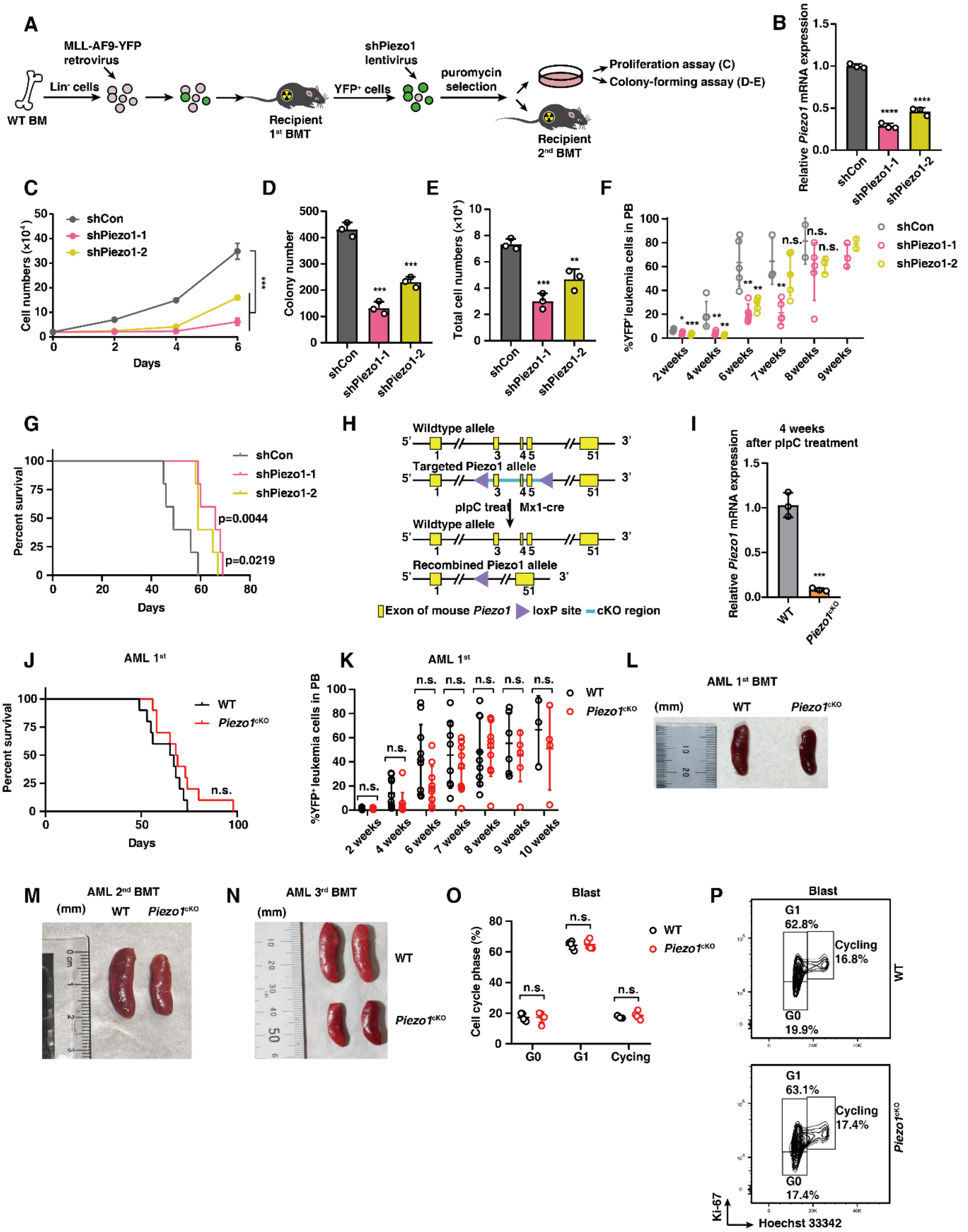

Supplementary Fig. S2 Piezo1 Is Required for Murine AML Development

(A) Experimental scheme for **(B-G)**.

(B) qRT-PCR showing knockdown efficiency of Piezo1 in mice leukemia cells. (n=3)

(C) Growth curves of mice leukemia cells after transduction with the indicated lentiviruses. (n=3) Two-way ANOVA with Sidak's post-hoc test.

(D-E) Colony formation assay of mice leukemia cells **(D)** and cell numbers **(E)** after transduction with the indicated lentiviruses. (n=3)

(F) Percentages of YFP^+ leukemia cells in PB at the indicated time after transplantation (n = 5 per group). Two-way ANOVA with Sidak's post-hoc test.

(G) Kaplan-Meier survival curves for recipient mice receiving equal number of YFP^+ leukemia cells after Piezo1 knockdown (n = 5 per group). Two-sided log-rank test.

(H) Scheme for generating *Piezo1*^{cKO} mouse.

(I) qRT-PCR analysis showing the efficiency of Piezo1 deletion. (n=3)

(J) Kaplan-Meier survival curves for recipients of MLL-AF9-transduced Lin- bone marrow cells from WT and *Piezo1*^{cKO} donor mice. (n=5 per group) Two-sided log-rank test.

(K) Percentages of YFP^+ leukemia cells in PB at the indicated time after transplantation. (n=5 per group) Two-way ANOVA with Sidak's post-hoc test.

(L) Representative images of the spleens from WT and *Piezo1*^{cKO} 1st BMT recipient mice.

(M-N) Representative images of the spleens from WT and *Piezo1*^{cKO} 2nd **(M)** and 3rd **(N)** BMT recipient mice.

(O) Cell cycle analysis showing percentages of cell cycle phases of blast cells from WT and *Piezo1*^{cKO} AML mice (n=5).

(P) Representative flow cytometry plot showing cell cycle analysis of blast cells from WT and *Piezo1*^{cKO} AML mice.

*p < 0.05, **p < 0.01, ***p < 0.001 and ****p < 0.0001. Unless otherwise specified in individual panels, all data are presented as mean ± SD (error bars), and statistical significance was determined using two-tailed unpaired Student's t-test (paired where indicated). Results are representative of two (**C-G, J-P**) and three (**B, I**) independent experiments.

Supplemental Figure 3

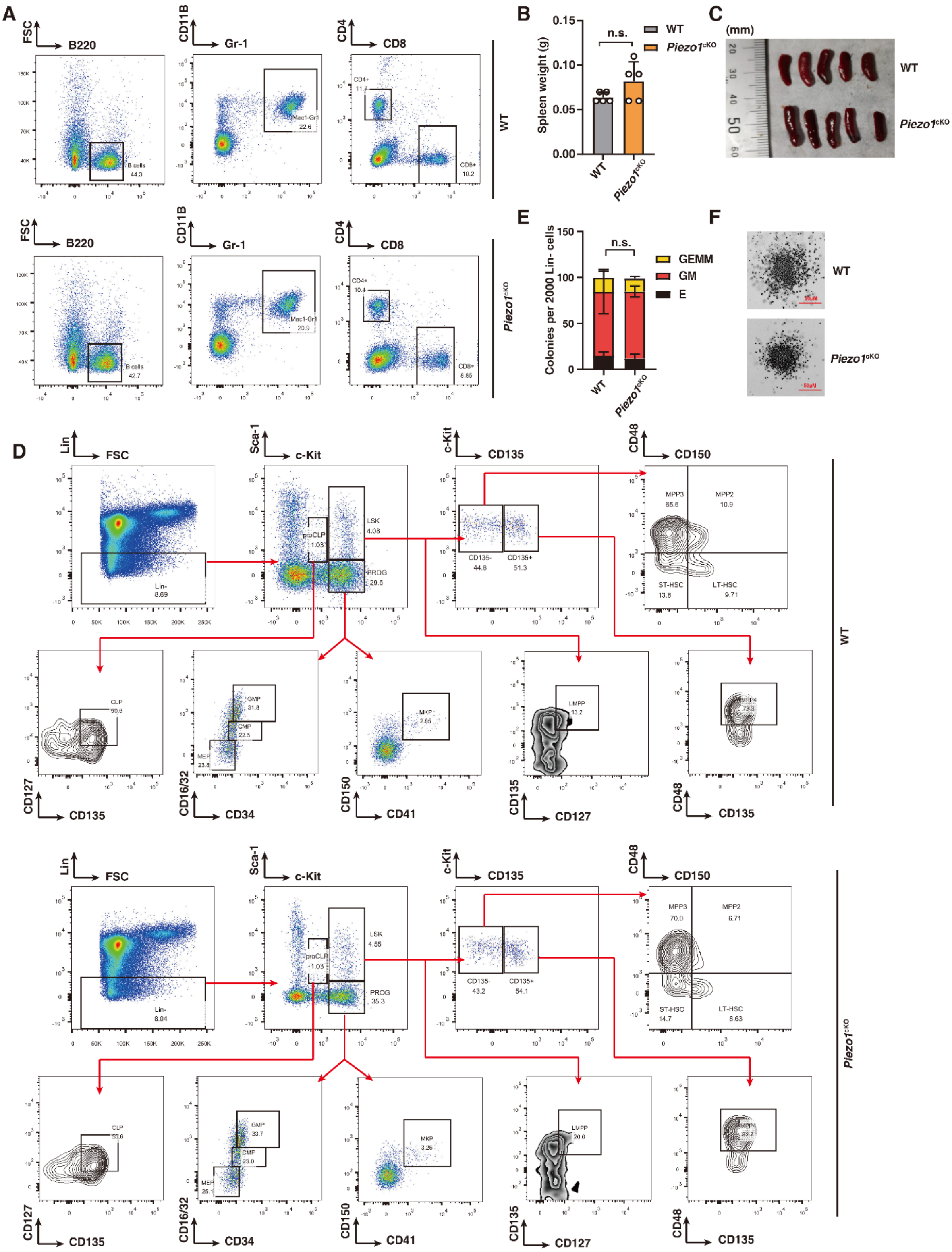

Supplementary Fig. S3 Deletion of Piezo1 Does Not Affect Murine Normal Hematopoiesis

(A) Representative FACS plots showing the gating strategy for different lineage cells in PB from WT and *Piezo1*^{cKO} mice.

(B) Spleen weight of WT and *Piezo1*^{cKO} mice (n = 5).

(C) Representative images of the spleens from WT and *Piezo1*^{cKO} mice.

(D) Representative FACS plots showing the gating strategy for different stem and progenitor cell populations in bone marrow from WT and *Piezo1*^{cKO} mice.

(E) CFU assay of 2000 Lin^{-} BM cells from WT and *Piezo1*^{cKO} mice.

(F) Images showing the representative colonies for WT and *Piezo1*^{cKO} mice.

*p < 0.05, **p < 0.01, ***p < 0.001 and ****p < 0.0001. Unless otherwise specified in individual panels, all data are presented as mean ± SD (error bars), and statistical significance was determined using two-tailed unpaired Student's t-test (paired where indicated). Results are representative of two independent experiments (**B, E**).

Supplemental Figure 4

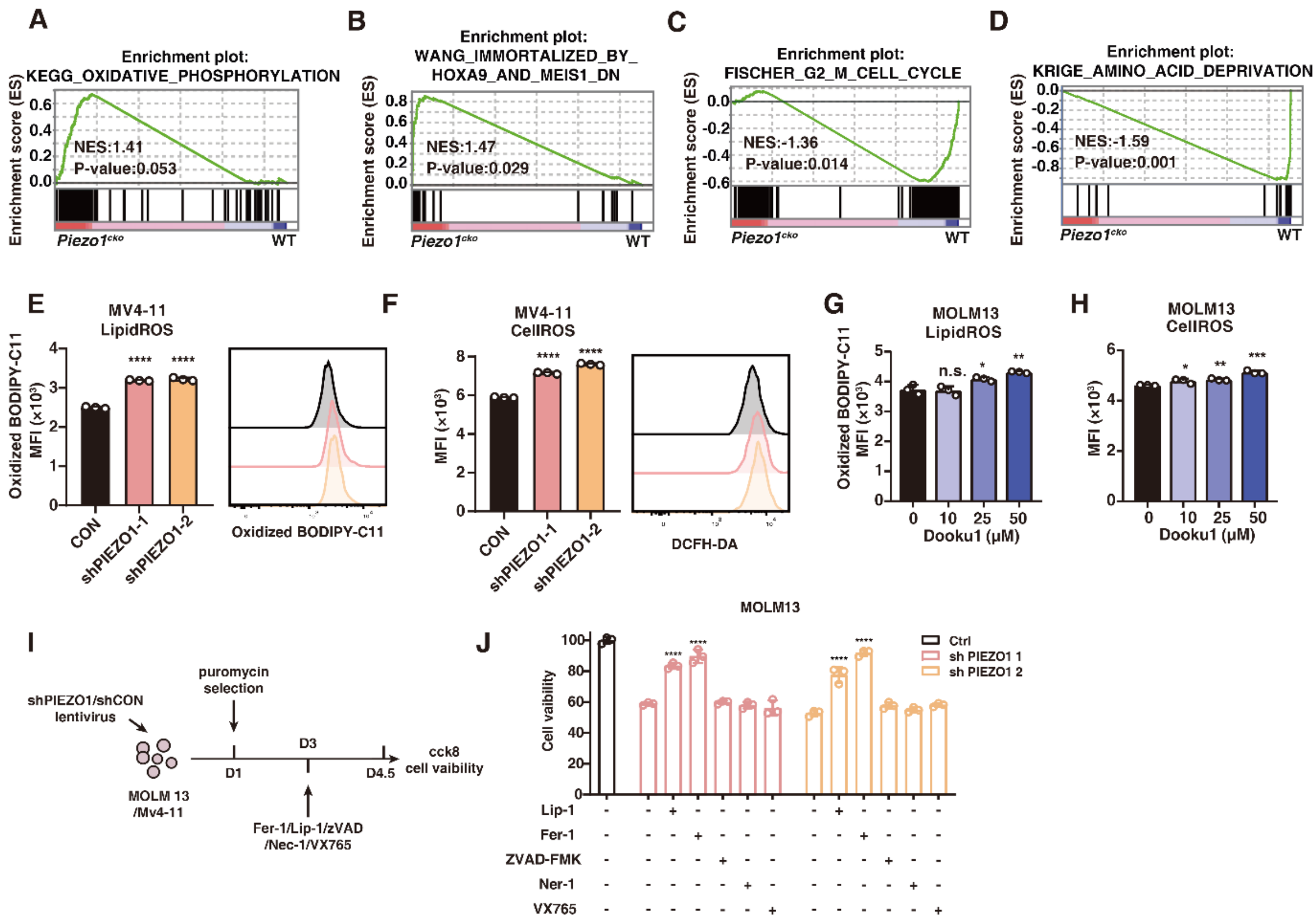

Supplementary Fig. S4 PIEZO1 Protected Leukemia Cells from Ferroptosis in vitro

(A-B) GSEA plot showing enrichment of gene sets of oxidative phosphorylation **(A)** and immortalized by HOXA9 and MEIS1 down **(B)** in *Piezo1*^{cKO} LSCs.

(C-D) GSEA plot showing enrichment of gene sets of G2-M cell cycle **(C)** and amino acid deprivation **(D)** in WT LSCs.

(E-F) Lipid peroxidation levels analysis **(E)** and ROS levels analysis **(F)** of MV4-11 leukemia cells after transduction with the indicated lentiviruses. (n=3) One-way ANOVA with Tukey's post-hoc test.

(G-H) Lipid peroxidation levels analysis **(G)** and ROS levels analysis **(H)** of MOLM13 leukemia cells treated with Dooku1 in different doses (0, 10, 25, 50 μM). (n=3) One-way ANOVA with Tukey's post-hoc test.

(I) Experimental scheme for **(J)**.

(J) Cell viability of MOLM13 cells expressing shPIEZO1 or control following treatment with cell death inhibitors for 36 h, as measured by CCK-8 assay. Fer-1 (5 μM), Lip-1 (5 μM), Z-VAD-FMK (20 μM), Nec-1s (10 μM), VX-765 (20 μM). n=3 independent experiments, each in triplicate. Two-way ANOVA with Tukey's post-hoc test.

*p < 0.05, **p < 0.01, ***p < 0.001 and ****p < 0.0001. Unless otherwise specified in individual panels, all data are presented as mean ± SD (error bars), and statistical significance was determined using two-tailed unpaired Student's t-test (paired where indicated). Results are representative of three independent experiments (**E-J**).

Supplementary Figure 5

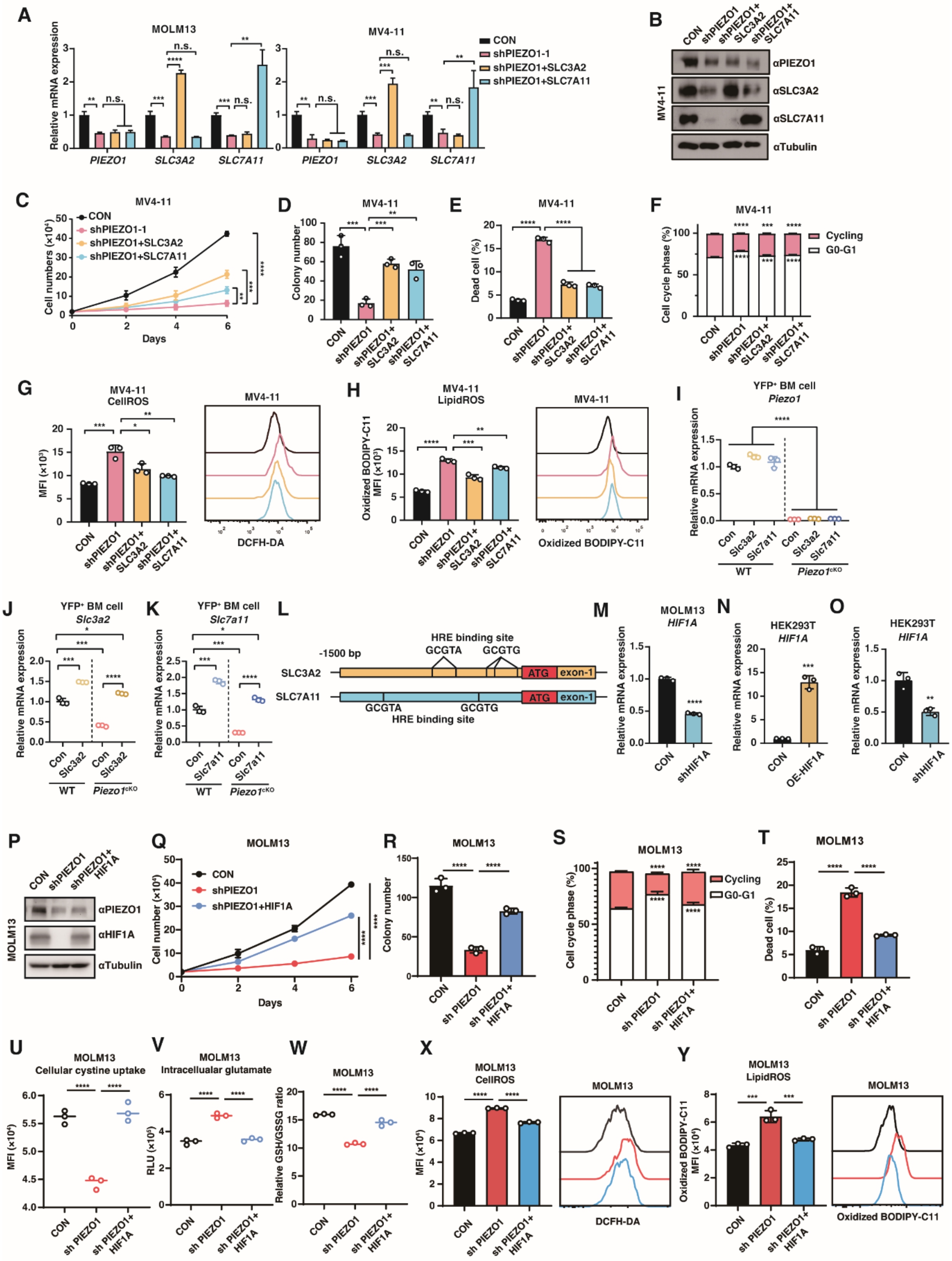

Supplementary Fig. S5 System Xc⁻ Is Required for AML Maintenance

(A) qRT-PCR showing expression of PIEZO1, SLC3A2 and SLC7A11 in MOLM13 (Left) leukemia cells and MV4-11 (Right) leukemia cells after transduction with the indicated lentiviruses. n=3 independent experiments. One-way ANOVA with Tukey's post-hoc test.

(B) Western blot assay showing expression of PIEZO1, SLC3A2 and SLC7A11 in MV4-11 leukemia cells. Tubulin served as the loading control.

(C-H) Growth curves **(C)**, CFU assay **(D)**, cell death analysis **(E)** and cell cycle distribution **(F)**, ROS levels analysis **(G)** and lipid peroxidation levels analysis **(H)** of MV4-11 leukemia cells after transduction with the indicated lentiviruses. n=3 independent experiments, each in triplicate (except CFU in duplicate). For (C), two-way ANOVA with Sidak's post-hoc test. For (D–H), one-way ANOVA with Tukey's post-hoc test.

(I-K) qRT-PCR showing expression of Piezo1 **(I)**, Slc3a2 **(J)** and Slc7a11 **(K)** in YFP^+ BM leukemia cells from WT and $Piezo1^{cKO}$ mice after transduction with the indicated lentiviruses. (n=3) Two-way ANOVA with Tukey's post-hoc test.

(L) Schematic diagram of HIF1A HRE binding site in SLC3A2 and SLC7A11 promoter.

(M) qRT-PCR showing expression of HIF1A in MOLM13 leukemia cells after transduction with the indicated lentiviruses.

(N-O) qRT-PCR showing expression of HIF1A in HEK293T cells after transduction with the indicated lentiviruses.

(**P**) Western blot assay showing expression of PIEZO1, SLC7A11 in MOLM13 leukemia cells. Tubulin served as the loading control.

(**Q-Y**) Growth curves (**Q**), CFU assay (**R**), cell cycle distribution (**S**), cell death analysis (**T**), cellular cystine uptake (**U**), intracellular glutamate level (**V**), GSH/GSSG ratio (**W**), ROS levels analysis (**X**) and lipid peroxidation levels analysis (**Y**) of MOLM13 leukemia cells after transduction with the indicated lentiviruses. n=3 independent experiments. For (Q), two-way ANOVA with Sidak's post-hoc test. For (R–Y), one-way ANOVA with Tukey's post-hoc test.
*p < 0.05, **p < 0.01, ***p < 0.001 and ****p < 0.0001. Unless otherwise specified in individual panels, all data are presented as mean ± SD (error bars), and statistical significance was determined using two-tailed unpaired Student's t-test (paired where indicated). Results are representative of three independent experiments (A-K, M-Y).

Supplemental Figure 6

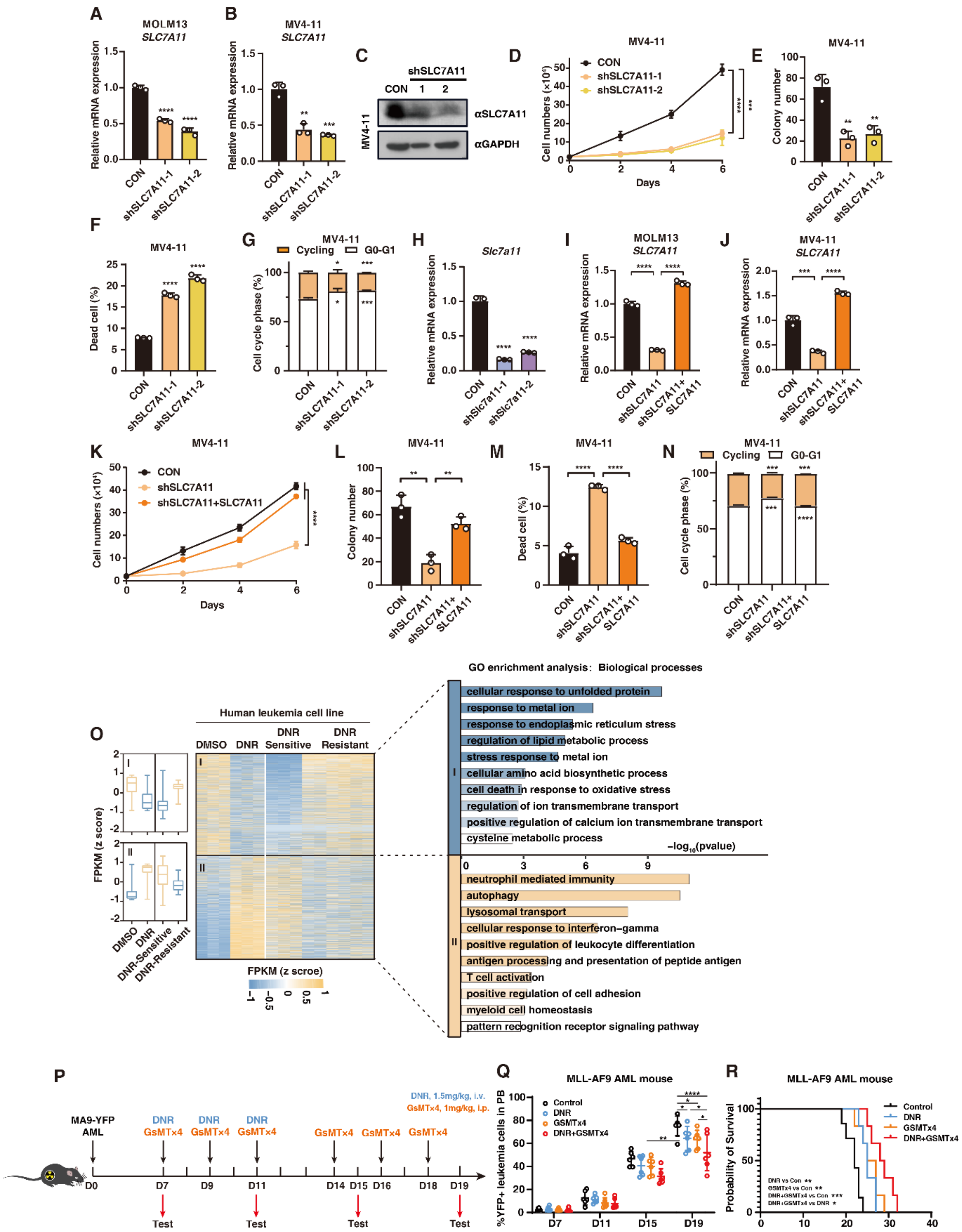

Supplementary Fig. S6 Inhibition of PIEZO1-SLC7A11 Axis Suppresses LSC Function and Contributes to Overcoming AML Chemoresistance

(A-B) qRT-PCR showing knockdown efficiency of SLC7A11 in MOLM13 **(A)** and MV4-11 **(B)** leukemia cells. (n=3)

(C) Immunoblot showing expression of SLC7A11 in MV4-11 leukemia cells after transduction with the indicated lentiviruses.

(D-G) Growth curves **(D)**, CFU assay **(E)**, cell death analysis **(F)** and cell cycle distribution **(G)** of MV4-11 leukemia cells after transduction with the indicated lentiviruses. n=3 independent experiments. For (D), two-way ANOVA with Sidak's post-hoc test. For (E-G), one-way ANOVA with Tukey's post-hoc test.

(H) qRT-PCR showing knockdown efficiency of Slc7a11 in mice leukemia cells. (n=3)

(I-J) qRT-PCR showing expression of SLC7A11 in MOLM13 **(I)** and MV4-11 **(J)** leukemia cells after transduction with the indicated lentiviruses. n=3 independent experiments. One-way ANOVA with Tukey's post-hoc test.

(K-N) Growth curves **(K)**, CFU assay **(L)**, cell death analysis **(M)** and cell cycle distribution **(N)** of MV4-11 leukemia cells after transduction with the indicated lentiviruses. n=3 independent experiments. For (K), two-way ANOVA with Sidak's post-hoc test. For (L-N), one-way ANOVA with Tukey's post-hoc test.

(O) The trend of mRNA dynamics (Left), heatmap (Middle) and GO enrichment analysis (Right) of differential gene expression in DMSO-treated, DNR-treated, DNR-sensitive and DNR-resistant human leukemia cells (GSE227269 and GSE131823). GO enrichment: Fisher's exact test with Benjamini-Hochberg correction; adjusted $P < 0.05$.

(P) Experimental scheme for **(Q-R)**.

(Q) Percentages of YFP^+ leukemia cells in PB of AML mice at the indicated time points following treatment with the specified compounds (n = 6 per group). Two-way ANOVA with Tukey's post-hoc test.

(R) Kaplan–Meier survival curves of recipient mice transplanted with equal numbers of YFP^+ leukemia cells and treated with the specified compounds (n = 6 per group). Two-sided log-rank test.

*p < 0.05, **p < 0.01, ***p < 0.001 and ****p < 0.0001. Unless otherwise specified in individual panels, all data are presented as mean ± SD (error bars), and statistical significance was determined using two-tailed unpaired Student's t-test (paired where indicated). Results are representative of three independent experiments (A-N).

Table S1 List of Primer sequences.

Name	Sequence 5'-3'
**Primers for RT-PCR**	
q-GAPDH-F	GGAGCGAGATCCCTCCAAAAT
q-GAPDH-R	GGCTGTTGTCATACTTCTCATGG
q-ACTB-F	GAGAAAATCTGGCACCACACC
q-ACTB-R	GGATAGCACAGCCTGGATAGCAA
q-PIEZO1-F	ATGTTGCTCTACACCCTGACC
q-PIEZO1-R	CCAGCACACACATAGATCCAGT
q-Gapdh-F	AGGTCGGTGTGAACGGATTTG
q-Gapdh-R	GGGGTCGTTGATGGCAACA
q-Piezo1-F	CCTGTTACGCTTCAATGCTCT
q-Piezo1-R	GTGTAGGCATATCTGAAAGGCAA
q-SLC3A2-F	GTGCTGGGTCCAATTCACAAG
q-SLC3A2-R	CACCCCGGTAGTTGGGAGTA
q-SLC7A11-F	GGTCCATTACCAGCTTTTGTACG
q-SLC7A11-R	AATGTAGCGTCCAAATGCCAG
q-ChIP-SLC3A2-NC-F	GGCGTAGACAAAGCGCCA
q-ChIP-SLC3A2-NC-R	AGGGTTAGAGGGTAGGCA
q-ChIP-SLC3A2-F	CACGAAGCCACTTACCACGA
q-ChIP-SLC3A2-R	GTCGCATGCGGTCAACGTA
q-ChIP-SLC7A11-NC-F	GTCTGAAAGCAGAGGAAG
q-ChIP-SLC7A11-NC-R	TTGCCCAGGGAAGGCAGC
q-ChIP-SLC7A11-F	TCCCTTCTTTCCCTGCCAAC
q-ChIP-SLC7A11-R	CCCTGAAGCTACCTTTATACGCA
q-HIF1A-F	GAACGTCGAAAAGAAAAGTCTCG
q-HIF1A-R	CCTTATCAAGATGCGAACTCACA
q-CP-F	GGGCCATCTACCCTGATAACA
q-CP-R	TTAAAGGTCCGATGAGTCCTGA
q-GCLM-F	CATTTACAGCCTTACTGGGAGG
q-GCLM-R	ATGCAGTCAAATCTGGTGGCA
q-PCBP1-F	AAAGGCGGGTGTAAGATCAAAG
q-PCBP1-R	GGCAAATCTGCTTGACACACTC
q-TFRC-F	TCGTGAGGCTGGATCTCAAAA
q-TFRC-R	CCTTACTATACGCCACATAACCC
q-ACSL3-F	ATGGAAAACCAACCTCATAGCAA
q-ACSL3-R	GCCATCCCAGTTATACCAGCAA
q-Slc3a2-F	TGATGAATGCACCCTTGTACTT
q-Slc3a2-R	GCTCCCCAGTGAAAGTGGA

q-Slc7a11-F	GGCACCGTCATCGGATCAG
q-Slc7a11-R	CTCCACAGGCAGACCAGAAAA
Primers for shRNA	
shPIEZO1#1	CTCACCAAGAAGTACAATCAT
shPIEZO1#2	GTACAACGTCACCGTCATCAT
shPiezo1#1	GGAGCTGGATGATGACGATGA
shPiezo1#2	GGATGATGACGATGACGATGA
shHIF1A	GCCGCTCAATTTATGAATATT
shSLC7A11#1	CCCTCTATTCGGACCCATTTA
shSLC7A11#2	GCACCCTTTGACAATGATAAT
shSlc7a11#1	TGGGTGGAACTGCTCGTAATA
shSlc7a11#2	TGGAGTTATACAGCTAATTAA

Supplementary Materials and Methods

Flow cytometry analysis and cell sorting

Bone marrow cells from mice were treated with an erythrocyte lysis solution on ice for five minutes to remove erythrocytes, followed by washing with PBS containing 2% FBS. The processed cells were subjected to cell surface marker staining.

For mouse HSPC and LSC studies, cell surface markers are as follows: LT-HSC ($Lin^-Sca1^+c\text{-}Kit^+CD135^-CD48^-CD150^+$); ST-HSC ($Lin^-Sca\text{-}1^+c\text{-}Kit^+CD135^-CD48^-CD150^{-}$); MPP2 ($Lin^-Sca\text{-}1^+c\text{-}Kit^+CD135^-CD48^+CD150^+$); MPP3 ($Lin^-Sca\text{-}1^+c\text{-}Kit^+CD135^-CD48^+CD150^-$); MPP4 ($Lin^-Sca\text{-}1^+c\text{-}Kit^+CD135^+CD48^+CD150^+$); LMPP ($Lin^-Sca\text{-}1^+c\text{-}Kit^+CD135^+CD127^+$); GMP ($Lin^-Sca\text{-}1^-c\text{-}Kit^+CD16/32^{hi}CD34^+$); CMP ($Lin^-Sca\text{-}1^-c\text{-}Kit^+CD16/32^{mid}CD34^+$); MEP ($Lin^-Sca\text{-}1^-c\text{-}Kit^+CD16/32^{low}CD34^-$); CLP ($Lin^-Sca1^{low}c\text{-}Kit^{low}CD135^+CD127^+$); LSC ($Lin^-Sca\text{-}1^-c\text{-}Kit^+CD16/32^{hi}CD34^+YFP^+$).

ChIP-PCR Relevant reagent formulations

Hypotonic Lysis buffer (25 mM HEPES (pH 7.9), 1.5 mM $MgCl_2$, 10 mM KCl, and 0.1% NP-40); IP buffer (20 mM Tris-Cl, pH 7.9; 150 mM NaCl; 1 mM EDTA; 1% Triton X-100; 0.1% Na-deoxycholate and 0.1% SDS); High-salt IP buffer (as above but with 500 mM NaCl); LiCl buffer (10 mM Tris-Cl, pH 7.9; 250 mM LiCl; 1 mM EDTA; 0.5% Na-deoxycholate; 0.5% NP-40); TE buffer (10 mM Tris-Cl, pH 7.9; 1 mM EDTA).
